# Comparison of hoop-net trapping and visual surveys to monitor abundance of the Rio Grande cooter (*Pseudemys gorzugi*)

**DOI:** 10.7717/peerj.4677

**Published:** 2018-05-11

**Authors:** Ivana Mali, Adam Duarte, Michael R.J. Forstner

**Affiliations:** 1 Department of Biology, Eastern New Mexico University, Portales, NM, USA; 2 Oregon Cooperative Fish and Wildlife Research Unit, Department of Fisheries and Wildlife, Oregon State University, Corvallis, OR, USA; 3 Department of Biology, Texas State University, San Marcos, TX, USA

**Keywords:** Capture–recapture, Abundance, *Pseudemys gorzugi*, Rio Grande cooter, Visual survey, Status, Distance sampling

## Abstract

Abundance estimates play an important part in the regulatory and conservation decision-making process. It is important to correct monitoring data for imperfect detection when using these data to track spatial and temporal variation in abundance, especially in the case of rare and elusive species. This paper presents the first attempt to estimate abundance of the Rio Grande cooter (*Pseudemys gorzugi*) while explicitly considering the detection process. Specifically, in 2016 we monitored this rare species at two sites along the Black River, New Mexico via traditional baited hoop-net traps and less invasive visual surveys to evaluate the efficacy of these two sampling designs. We fitted the Huggins closed-capture estimator to estimate capture probabilities using the trap data and distance sampling models to estimate detection probabilities using the visual survey data. We found that only the visual survey with the highest number of observed turtles resulted in similar abundance estimates to those estimated using the trap data. However, the estimates of abundance from the remaining visual survey data were highly variable and often underestimated abundance relative to the estimates from the trap data. We suspect this pattern is related to changes in the basking behavior of the species and, thus, the availability of turtles to be detected even though all visual surveys were conducted when environmental conditions were similar. Regardless, we found that riverine habitat conditions limited our ability to properly conduct visual surveys at one site. Collectively, this suggests visual surveys may not be an effective sample design for this species in this river system. When analyzing the trap data, we found capture probabilities to be highly variable across sites and between age classes and that recapture probabilities were much lower than initial capture probabilities, highlighting the importance of accounting for detectability when monitoring this species. Although baited hoop-net traps seem to be an effective sampling design, it is important to note that this method required a relatively high trap effort to reliably estimate abundance. This information will be useful when developing a larger-scale, long-term monitoring program for this species of concern.

## Introduction

Natural resource agencies rely on monitoring programs to facilitate state-dependent decision making when managing animal populations (Duarte et al., 2017; Nichols et al., 2017; O’Donnell et al., 2017). In the case of species of concern, abundance is often of interest because it offers information on the current status of the species and can be used to evaluate the effectiveness of alternative management actions. However, indices of relative abundance (i.e., minimum known alive, capture per unit effort (CPUE), etc.) are often complicated by sampling bias and variance. Indeed, the ability to capture individuals in a population can be related to an individual’s size, sex, and behavior, bait preferences, and the habitat characteristics of the sampling area, among other factors (Mali et al., 2014a; Chauvenet et al., 2017; Keiter et al., 2017; de Oliveira e Silva et al., 2017). This requires managers to develop sampling designs that allow them to distinguish true spatial and temporal variation in abundances from variation in sampling efficiency (i.e., detection/capture probability) so that monitoring data may appropriately inform management decisions (MacKenzie & Kendall, 2002).

The Rio Grande cooter (*Pseudemys gorzugi*) is a relatively large riverine turtle that is native to the Lower Rio Grande River Basin and its tributaries (Pierce et al., 2016). The species is listed as threatened in Mexico and New Mexico (New Mexico Department of Game and Fish [NMDGF], 2006; Secretaríade Medio Ambiente y Recursos Naturales, 2010) and a Species of Greatest Conservation Need in Texas (Texas Parks and Wildlife Department [TPWD], 2012), concurrent to its review for federal protection by the United States Fish and Wildlife Service (reviewed in Pierce et al., 2016). Human modification of habitats and ongoing detrimental population-level effects of historic commercial harvest represent potential threats to the species (Dixon, 2013; Mali et al., 2014b; Pierce et al., 2016). Unfortunately, very little is known about this turtle’s demography, ecology, and natural history, and there is an increasing need for information on population composition and abundance to further existing conservation measures (Ernst & Lovich, 2009; Lovich & Ennen, 2013; Pierce et al., 2016). Current research suggests Rio Grande cooters generally prefer sections of river with deep clear pools (Degenhardt, Painter & Price, 1996), but the species has also been found in nearby lentic water bodies (reviewed in Pierce et al., 2016). While the species has been found to be locally abundant at a few locations (Dixon, 2013), a low range-wide population density may be natural for the species (Bailey et al., 2008); however, no attempts have been made to estimate Rio Grande cooter distribution and abundance while accounting for imperfect detection.

The lack of robust demographic estimates for this species is, in part, related to the fact that *Pseudemys* turtles are generally more difficult to survey than most freshwater turtle species. The most common survey technique (i.e., baited hoop-net traps) can be unsuccessful because *Pseudemys* turtles are predominantly herbivorous as adults (Lindeman, 2007). Oddly, baited hoop-net traps have been a successful survey technique for Rio Grande cooters at sites in New Mexico, but not in Texas (Degenhardt, Painter & Price, 1996). Sterrett et al. (2010) found that snorkel surveys yielded higher counts for river cooter (*Pseudemys concinna*) than baited hoop-net traps. Conversely, in a concurrent study we have found snorkel surveys to be limited by water clarity, particularly in the low-visibility water bodies of New Mexico. In 2016, our snorkel surveys yielded no turtles in the lower stretches and only four turtles in the upper stretches of the Black River, New Mexico. MacCulloch & Gordon (1978) suggested basking traps may be a more effective survey technique for *Pseudemys* turtles because they typically bask in large numbers during summer months. Analogously, Bailey et al. (2008) were successful in conducting visual surveys of Rio Grande cooters as they basked on the river bank and woody debris floating in the water.

Given the conservation status of Rio Grande cooter and scarcity of available information, it is prudent to assess their current status and identify optimal survey techniques for short- and long-term monitoring programs to help facilitate informed management and policy decisions. In this paper, we compared the efficacy of traditional capture–recapture surveys via baited hoop-net traps to less invasive visual surveys to estimate abundance while accounting for imperfect detection.

## Methods

### Study site

**Figure 1:**
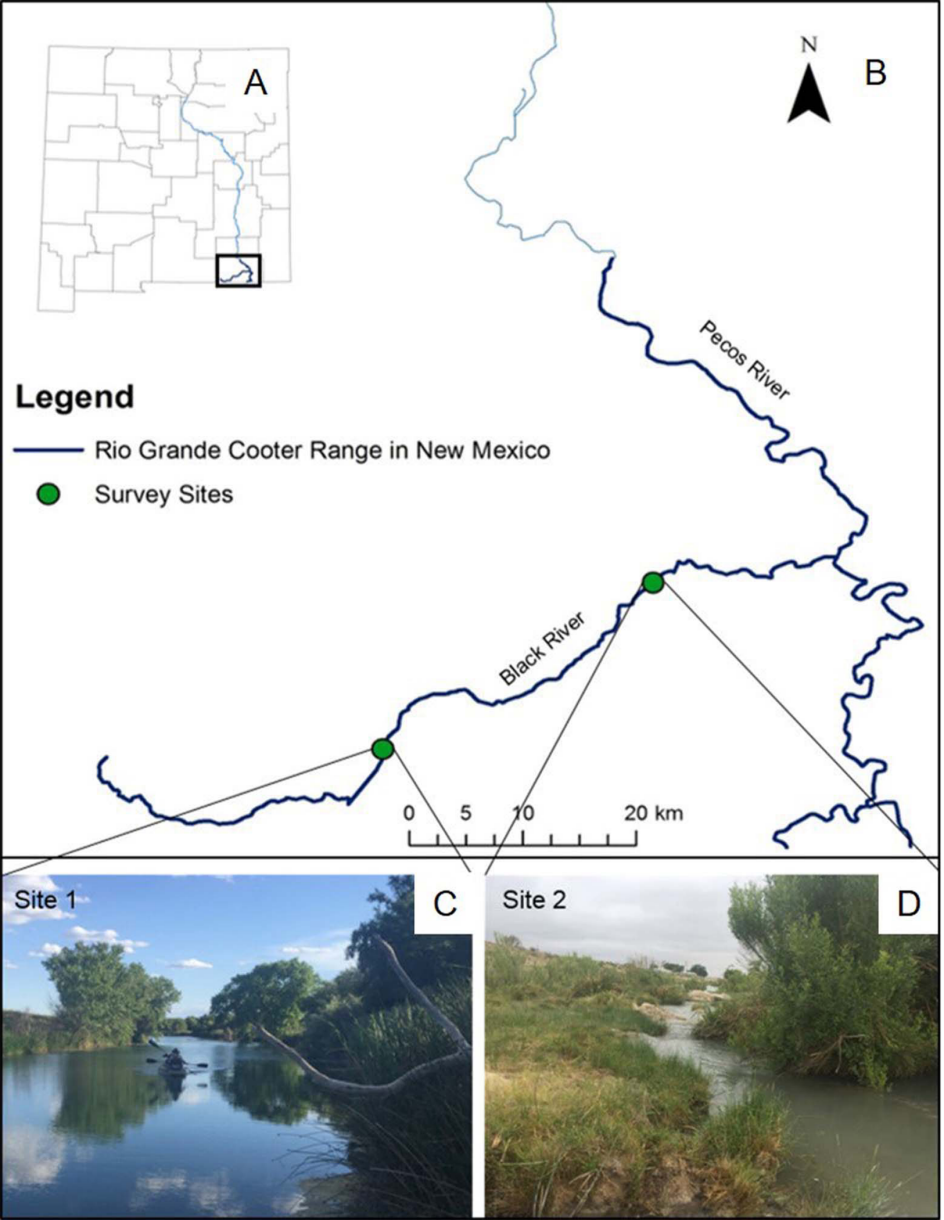
Rio Grande cooter survey sites. A map of New Mexico (A) and specifically the range of Rio Grande cooter (*Pseudemys gorzugi*) south of Avalon Dam on the Pecos River and its tributaries (B). Figure depicts two survey sites along the Black River showing different habitat characteristics. The upstream site 1 (C) represents wide and interrupted stretch of the river while the downstream site 2 (D) often contains narrow riffles. Photographs of the sites were taken by I. Mali.

The Pecos River is a major tributary of the Lower Rio Grande Basin, where populations of Rio Grande cooter occur below Avalon Dam. Our study occurred along the Black River, which is a ∼87 km long tributary of the Pecos River, located in Eddy County, New Mexico. Land uses surrounding the Black River include cattle ranching and oil extraction, but human water use is predominantly for irrigation practices. Although the river is primarily on the surface, the river can also run underground for several km at a time. We surveyed two stretches of the river (Fig. 1). The upper stretch of river (site 1) is ∼1.5 km long and ∼20 m wide, for a total effective sampling area of ∼30,000 m^2^. This section of the river is located in close proximity to headwaters of the Black River and managed by the Bureau of Land Management (BLM). Here, water depth and visibility varies from 1.9 to 3.8 m and from 0.8 to 2.5 m, respectively. This site is particularly unique because of its abundance of basking habitats, particularly felled trees in the river. Rio Grande cooters have been captured via baited hoop-net traps and observed basking in large numbers at this site (Degenhardt, Painter & Price, 1996), so we were certain of the historic presence of the species. We surveyed another ∼1.5 km stretch of river located ∼30 km downstream from site 1. This site (site 2) has a variable river width (2 to 50 m), for a total effective sampling area of ∼34,500 m^2^. Site 2 is located within private property and has not been surveyed previously. Water at this site is shallower, ranging from 1.0 to 2.5 m, and visibility ranges from 0.3 to 0.9 m.

While at site 1 we used both baited hoop-net traps and visual surveys, we only used baited hoop-net traps at site 2 due to physical characteristics of the habitat. Specifically, site 2 lacks available basking habitats (i.e., large rocks, felled trees, or gentle bank slopes) and consists of a couple of waterfalls and narrow shallow regions that required us to exit the boat and portage, which alerted nearby turtles and prevented us from obtaining accurate observational records. Therefore, we were not able to effectively conduct visual surveys at site 2.

### Capture–recapture surveys

We used single-opening, wide-mouth hoop-net traps with 76.2 cm diameter and 2.54 cm mesh (Memphis Net & Twine Co. Memphis, Tennessee, USA) baited with shrimp and sardine-based baits (Degenhardt, Painter & Price, 1996). Traps were positioned with the mouth facing downstream and bait was replaced every other day. Due to logistical constraints (i.e., number of available traps and river access points), site 1 was divided into two adjacent sections and site 2 was divided into three adjacent sections. We trapped each of these sections for six days using 50 traps, but some trap theft did occur (i.e., two traps were stolen at site 1 and four traps at site 2). We trapped site 1 from 23 May to 4 June 2016, resulting in 594 trap days (i.e., 1 trap day = 24 h period). We trapped site 2 from 13 June to 19 June and from 11 July to 23 July 2016, resulting in 874 trap days. The overall trap density was 70 per river kilometer at site 1 and 100 per river kilometer at site 2. Every trap remained on station for 6 days, and soak-time, or the interval between trap set and trap check was 24 h.

Each captured turtle was marked in one or more of the following ways: by notching marginal scutes (adults only; Cagle, 1939), by inserting passive integrated transponder tags (adults and juveniles; Buhlmann & Tuberville, 1998), or by toe clipping (small turtles only). We also photographed the plastron of hatchling turtles to identify individual recaptures. We conducted this research under NMDGF permit authorization #3621. Trapping and handling methods were approved by the Eastern New Mexico University Institutional Animal Care and Use Committee (Protocol No. 03-02/2016).

### Visual surveys

Visual surveys consisted of a person steering the boat from the stern while another person searched for basking turtles through binoculars from the bow. Transect line was the stream center line. The GPS coordinates were recorded and perpendicular distance of the basking turtle from the transect line was visually estimated for each encountered turtle. Because the number of turtles basking (i.e., number of turtles available for detection) can vary, we repeated our visual surveys (*N* = 6) at site 1 during the months of highest *P. gorzugi* activity (Degenhardt, Painter & Price, 1996). Five surveys occurred from 13 June to 18 June and one survey occurred on 8 August 2016. We selected days when the weather was ideal for turtle basking (i.e., sunny and warm), and the time of day the surveys took place varied from 0845 to 1530 h. We also classified each observed turtle as either a juvenile (<65 mm in straight line carapace length) or adult, where subadults larger than juveniles were grouped with adults. For consistency and accuracy of observations and age classifications, we always used the same trained observer during visual surveys (I. Mali who has >7 years of experience working with riverine turtles).

### Statistical analyses

#### Capture–recapture analyses

We analyzed the trap data using the model of Huggins (1989, 1991). This approach assumes the target population does not have turnover of individuals due to deaths, births, and movement in and out of the study area (i.e., population closure). Given the relatively short trapping session compared to the life span of *Pseudemys* turtles and sedentary nature of Rio Grande cooters in this river system (i.e., seasonal movement of 300 m; Degenhardt, Painter & Price, 1996), we are confident we meet the population closure assumption. We fitted the model to trap data collected at both sites together; however, juvenile and adult (including subadult) data were analyzed separately because age-specific differences in behavioral patterns that influence capture probabilities are likely dramatic. Capture–recapture data from all hoop-net traps set at a site on a given trap day were combined for each age class and treated as a single capture occasion. This is common practice when fitting this type of model to trap data, given trap captures probably lack independence. That is, capturing an individual in a given trap means that the individual is not available to be captured in any nearby traps on a given trap day and individual movement among traps between trap days is likely.

This estimator can model capture probabilities as a function of covariates. When fitting the models, the probability of first capture was allowed to differ from the probability of recapture (i.e., the probability of capturing a marked turtle). This pattern can be related to an assortment of reasons, particularly a change in the behavior of an individual following capture and marking events, and is a typical pattern found in capture–recapture data of aquatic species (Peterson, Thurow & Guzevich, 2004; Peterson, Scheerer & Clements, 2015; Duarte et al., 2017). We also included an intercept adjustment so that capture probability could differ between the sites (i.e., an additive model) to account for seemingly different habitats and possible differences in trap efficiency due to differing trap densities. For each analysis, we estimated abundance $({\hat N}_{\rm{CR}})$ and density for each site separately.

Model fitting used *WinBUGS* software version 1.4 (Spiegelhalter, Thomas & Best, 2006) called from program *R* (R Core Team, 2016) with package *R2WinBUGS* (Sturtz, Ligges & Gelman, 2005). Diffuse priors Normal (μ = 0, τ = 0.37), were used for intercepts and coefficients of the logistic regression used to model capture probabilities. Each model run consisted of three independent chains of 250,000 iterations with a burn-in of 125,000 iterations. Model convergence was assessed by visual examination of trace plots and the $\hat R$ diagnostic (Brooks & Gelman, 1998), and convergence $(\hat R < 1.01)$ was obtained for all parameter estimates. Posterior distributions were described by their mean and 95% credible interval (CRI). Furthermore, odds ratios were calculated for parameter estimates associated with capture probability to facilitate interpretation (Hosmer & Lemeshow, 2000).

#### Distance sampling

Visual survey data were analyzed using distance sampling methods. While the underlying principles of distance sampling are thoroughly covered by Buckland et al. (2001), the main assumptions are that the animals on the transect line have detection probability of 1, animals are detected at their initial location, and perpendicular distances of animals from the transect line are measured accurately. We binned the horizontal distances off the transect line at every 2 m resulting in five distance classes: 0–2, 2–4, 4–6, 6–8, and 8–10 m. To account for imperfect detection, we used the multinomial-Poisson mixture model (Royle, Dawson & Bates, 2004) implemented in the function *distsamp* in the package *unmarked* (Fiske & Chandler, 2011) called from program *R* (R Core Team, 2016). We used half-normal detection function because it yielded the lower AIC_c_ values in comparison to the model with hazard-rate detection function. As the density is returned in animals/ha on the log-scale, we back transformed the density and detection probability estimates, using the delta method to approximate standard errors and 95% confidence intervals (CI; Chandler, 2017). We derived the abundance estimate $({\hat N}_{\rm{DS}})$ based on the effective sampling area and the estimated density of turtles. To describe uncertainty of ${\hat N}_{\rm{DS}}$, we used a parametric bootstrap approach with 1,000 iterations (Chandler, 2017).

Given visual surveys were conducted in a short time span (i.e., within a season), we assumed that the actual turtle abundance did not change but that the variation in the number of turtles encountered is the result of differences in availability of turtles for detection (i.e., turtles present above the surface of the water). Although temporary emigration or availability for detection can be modeled, we did not have *a priori* hypotheses concerning what might influence this process. Estimating this additional parameter would require using a more complex model structure that would require more data to fit the model, and the aim of this study was restricted to the evaluation of the efficacy of using visual surveys to estimate abundance. Instead of approaching this potential problem using a statistical model, we attempting to address this potential problem through our sampling design. In particular, visual surveys were conducted when air temperatures were similar and ideal for basking behavior, visual survey days were close together, and all visual surveys were conducted during the peak basking period for the species and using the same observer. Thus, we fitted models to data collected during each of the six surveys separately and compared each of these estimates to the estimates from the capture–recapture model. Similar to our capture–recapture method, we analyzed juvenile and adult (which includes subadults) data separately.

## Results

We captured a total of 63 Rio Grande cooters at site 1, four of which were recaptures. At site 2, we captured a total of 157 Rio Grande cooters, 38 of which were recaptures. CPUE, calculated by dividing the total number of captures by total number of trap days, was 0.11 at site 1 and 0.18 at site 2. This metric provides some information about the trap effort required to obtain sufficient captures to enable capture–recapture analyses. During visual surveys, we observed between 18 and 44 turtles during a single survey event. The duration of a visual survey varied from 55 to 80 min and temperatures varied from 32 to 38 °C; there appeared to be no relationship between temperature and number of turtles observed during each survey (Table 1).

**Table 1:** A summary of six visual surveys of Rio Grande cooter (*Pseudemys gorzugi*).

Survey No.	Date	Time In	Time Out	Duration (h)	Temperature (°C)	No. turtles observed
1	6/13/2016	1530	1630	1	38	18
2	6/14/2016	1035	1150	1.25	34	41
3	6/16/2016	1030	1135	1.1	37	38
4	6/17/2016	0845	0940	0.92	32	19
5	6/18/2016	0930	1045	1.25	32	44
6	8/6/2016	1100	1220	1.33	32	22

**Note:**

A summary of six visual surveys of Rio Grande cooter (*Pseudemys gorzugi*) conducted in 2016 along the 1.5 km long stretch of the Black River, New Mexico (site 1). The surveys consisted of a person steering the boat from the stern while another person searched for basking turtles through binoculars from the bow. The fifth survey yielded the greatest number of turtles observed and was used for comparison to capture–recapture survey method.

### Capture–recapture analyses

The estimated turtle density differed between the sites, with 22.33 (SD = 2.31) adult turtles/ha (${\hat N}_{\rm{CR}}$ = 67, 95% CRI = 49–110) and 5.82 (SD = 0.33) juvenile turtles/ha (${\hat N}_{\rm{CR}}$ = 18, 95% CRI = 15–25) at site 1, and 37.10 (SD = 3.34) adult turtles/ha (${\hat N}_{\rm{CR}}$ = 128, 95% CRI = 96–211) and 10.68 (SD = 0.52) juvenile turtles/ha (${\hat N}_{\rm{CR}}=37$, 95% CRI = 32–51) at site 2. For juvenile turtles, the odds of recapturing an individual turtle was 1.55 times lower than the odds of capturing that same individual turtle for the first time, although this effect was not strong since the 95% CRI overlapped 0 (Table 2). As expected, the capture probability differed between the sites with the juvenile turtles having an 8.00 times lower odds of being captured at site 1 than at site 2. Similar patterns in capture probabilities were found for adult turtles (Table 2). Specifically, the odds of recapturing an individual adult turtle was 2.72 times lower than the odds of capturing that same individual turtle for the first time, and the odds of capturing an adult turtle was 5.47 times lower at site 1 than at site 2. Also, the odds of capturing an individual turtle was 2.03 times higher for juveniles than it was for adults.

**Table 2:** Model intercepts and coefficients for capture probabilities of Rio Grande cooter (*Pseudemys gorzugi*).

	Juveniles				Subadults/adults			
Parameter	Estimate	SD	Lower CRI	Upper CRI	Estimate	SD	Lower CRI	Upper CRI
Intercept	−0.73	0.39	−1.61	−0.09	−1.44	0.38	−2.31	−0.86
Recapture	−0.44	0.41	−1.21	0.42	−1.00	0.42	−1.77	−0.10
Site 1	−2.08	0.78	−3.74	−0.69	−1.70	0.65	−3.08	−0.54

**Note:**

Model intercepts and coefficients, including standard deviation (SD) and 95% credible intervals (CRI) for capture probabilities on logit scale based a closed capture–recapture analysis of Rio Grande cooter (*Pseudemys gorzugi*) along Black River, New Mexico. Note that the initial capture event and site 2 is the reference category.

### Distance sampling analyses

**Figure 2:**
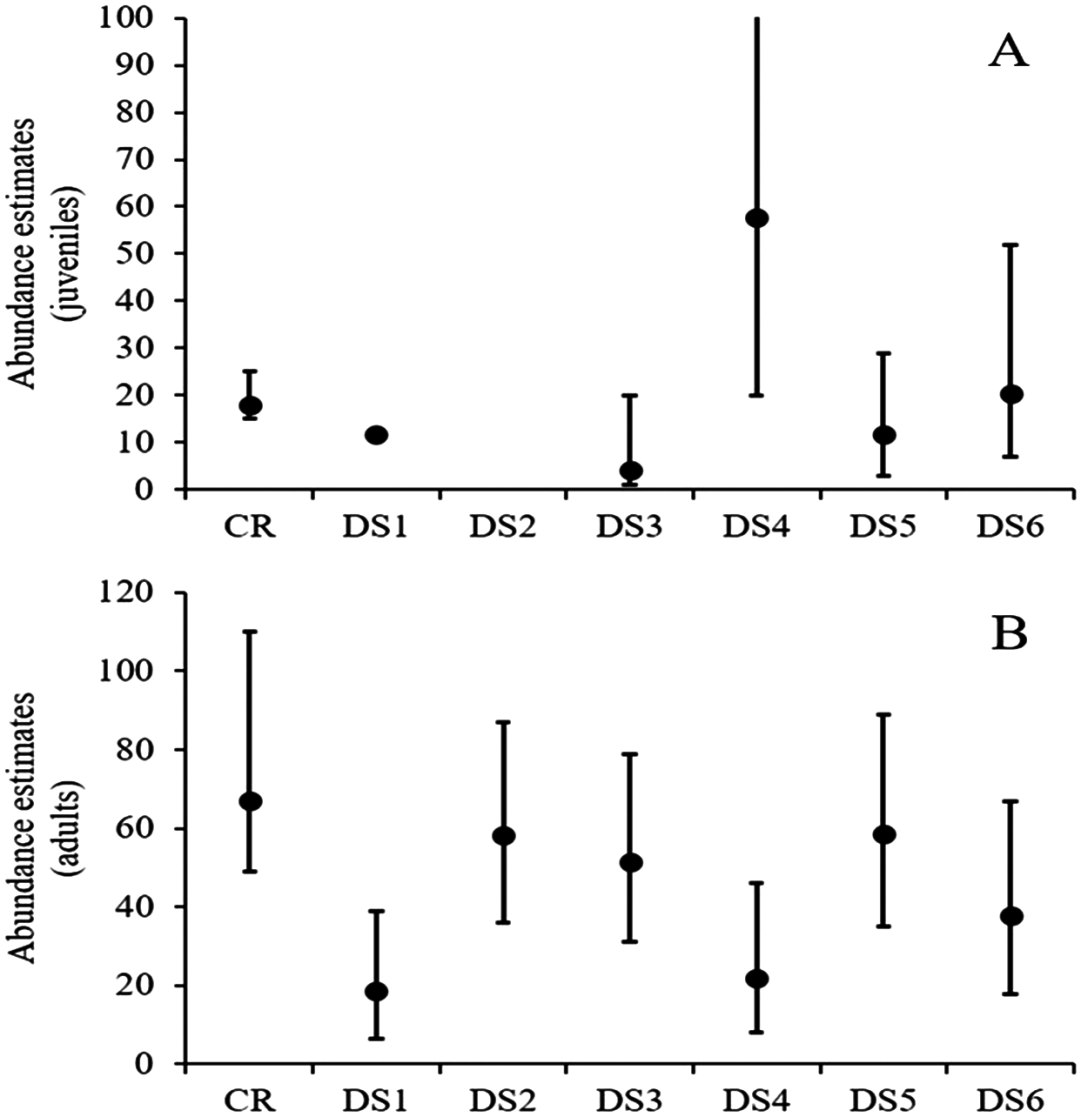
Rio Grande cooter abundance estimates. Derived Rio Grande cooter (*Pseudemys gorzugi*) abundance estimates for juveniles (turtles <65 mm straight carapace length; (A) and adults (including subadult turtles larger than juveniles; (B) with credible and confidence intervals using capture–recapture (CR) and six distance sampling events (DS1–DS6) at site 1 along the Black River, New Mexico. Note that in the case of juvenile turtles, for the first distance survey the data could not be converted and for the second distance survey event the data were too sparse to estimate abundance.

At site 1, density estimates ranged from six to 20 animals/ha for adults and from one to 19 animals/ha for juveniles, while estimated abundances varied from 19 to 59 for adults and four to 58 for juveniles (Fig. 2; Table 3). The fifth visual survey on 18 June 2016 yielded the highest number of observed turtles (*N* = 44). In this survey, the density of Rio Grande cooters was 19.6 (SE = 4.33) animals/ha and 3.94 (SE = 1.98) animals/ha for adults and juveniles, respectively. Derived abundance estimates were 59 (95% CI [36–87]) and 12 (95% CI [3–29]) for adults and juveniles, respectively. Detection probability declined with increasing distance from the transect line similarly for both age classes, with σ = 5.36 (SE = 1.1) for the adults and σ = 4.94 (SE = 2.09) for juveniles. Although this survey yielded similar estimates to capture–recapture analyses for both adults and juveniles, the remaining surveys resulted in highly variable estimates. Furthermore, the remaining distance sampling estimates were lower for adults and juveniles (with one exception for juveniles; Fig. 2; Table 3).

**Table 3:** Abundance estimates for visual distance sampling surveys of Rio Grande cooter (*Pseudemys gorzugi*).

	Juveniles				Subadults/Adults			
Survey No.	*D* (SE)	*N̂*	Lower CI	Upper CI	*D* (SE)	*N̂*	Lower CI	Upper CI
1	3.9 (2.0)	11.8	12	12	6.2 (2.7)	18.6	6.4	39
2	NA	NA	NA	NA	19.4 (4.3)	58.2	36	87
3	1.3 (0.9)	4.02	1	20	17.1 (4.0)	51.4	31	79
4	19.2 (8.4)	57.7	20	1003	7.3 (2.9)	21.9	8	46
5	3.9 (2.0)	11.8	3	29	19.6 (4.3)	58.7	35	89
6	6.8 (3.0)	20.4	7	52	12.6 (4.2)	37.8	18	67

**Note:**

Density estimates in animals/ha including standard error and abundance estimates with 95% confidence intervals for juvenile and adult (including subadult) Rio Grande cooters (*Pseudemys gorzugi*) for each of the six visual distance sampling surveys conducted in the summer 2016 along the Black River, New Mexico (site 1). Note that in the case of juvenile turtles, for the first survey the data could not be converted and for the second survey event the data were too sparse to estimate abundance.

## Discussion

Identifying effective methods for monitoring animal populations is essential to assess species’ status and properly inform policy and management decisions, especially in the case of species of conservation concern (Somers & Mansfield-Jones, 2008; Walker, 2012; Ebner et al., 2015). Rio Grande cooter has been a long overlooked freshwater turtle species of the American Southwest and there is a paucity of published data reporting the species’ demographic parameters or natural history traits (Lovich & Ennen, 2013; Lovich et al., 2016; Pierce et al., 2016). This is the first attempt to provide information on abundances while accounting for imperfect detection and the first evaluation of different sampling designs for this under-surveyed species. We explored the utility of a non-traditional, less labor intensive, and lower cost visual surveys to estimate abundance, and we were able to directly compare these estimates to estimates from more field intensive baited hoop-net trapping. Overall, we found that high intensity surveys via baited hoop-net traps can yield sufficient captures to enable estimates of abundance, while the abundance estimates using the visual survey data were inconsistent and generally lower.

Among chelonian research, distance sampling techniques have been used to estimate densities of tortoises (Swann, Averill-Murray & Schwalbe, 2002; Smith et al., 2009) and sea turtles (Gómez de Segura et al., 2003), but we are not aware of any published literature on distance sampling used for estimating abundances of riverine turtles. We found that only one abundance estimate from distance sampling protocols were congruent with estimates from a more intensive and traditional capture–recapture approach for riverine turtles, albeit the uncertainty associated with the point estimates was larger (Fig. 2). We found visual surveys could not be successfully employed at both of our sites, which may limit the usefulness of this method across a greater number of sites. For this technique to be useful, first, relatively long uninterrupted sections of the river are necessary to minimize disturbance of basking turtles. Second, the surveyed river section must provide appropriate basking habitats, such as rocks, felled trees, or thick floating vegetation that enable visual observation of the basking turtles.

Our replicate surveys at a single site show that the number of observed turtles can vary markedly within a short time period even under similar environmental conditions. It seems basking behavior of riverine turtles varies within a season. For example, in the cooler months or when temperatures are just starting to rise (i.e., early spring or summer mornings) turtles are almost strictly basking on structures laying above water, while in the hot summer months they are also basking among available herbaceous vegetation (i.e., subaerial basking). It is also important to be aware that basking habitats are subject to constant change due to high river flow events. Such seasonal patterns of turtle basking behavior and basking habitat should be considered if establishing monitoring programs centered on the use of visual surveys.

We acknowledge that our study is only a preliminary evaluation of visual surveys to estimate abundance via distance sampling for freshwater turtles and lacks replication. This makes it difficult to conclude whether the differences in abundance estimates using the different survey methods are typical or unusual. Thus, we recommend the efficacy of visual surveys to estimate abundance be further explored given traditional labor intensive and more expensive trapping methods are impractical in some areas due to difficult site access (i.e., remote areas), lack of resources (i.e., funding), and trap theft. If visual surveys are employed, we recommend replicate visual surveys be conducted at each site to maximize the number of turtles observed within a single survey. Notably, conducting replicate visual surveys is still less labor intensive than trapping turtles using baited hoop-net traps. For example, our six visual surveys resulted in ∼24 person hour in comparison to ∼156 person hour spent on hoop-net trapping at site 1.

We demonstrated that baited hoop-net traps remain a reliable method of surveying *P. gorzugi* on the Black River. Historic field surveys for this species on the Black River using baited hoop-net traps were done inconsistently in 1992, 1993, 2000, 2006, and 2007. For example, 1992 surveys resulted in 76 trap hours, 1993 surveys resulted in 284 trap hours, and 2000 surveys resulted in 19 trap hours. Combined, a total of 39 *P. gorzugi* were captured (C. W. Painter, 1992, 1993, 2000, unpublished data). During 2006 and 2007, 24 sites along the Black, Delaware, and Pecos Rivers were surveyed with a total effort of ∼212 trap days and a total of 52 *P. gorzugi* were captured (B. L. Christman & L. K. Kamees, 2017, unpublished data). Importantly, these lower overall sampling efforts (i.e., lower trap hours) did not allow us to retroactively correct the capture data for sampling efficiency using our estimates of capture probability. Given our study demonstrates high variability in capture probabilities, it is possible the higher CPUE reported in historic data are related to higher abundances, higher capture efficiencies, or both.

Our results are particularly unique because of the relatively high hatchling capture and detection probabilities via baited hoop-net traps and visual observations, respectively. It is worth pointing out that naïve hatchlings were not as frightened by the observer boat in comparison to the adults that sometimes fled to the water even at large distances from the observer (>30 m). The fact that hatchlings were more likely to be captured in baited hoop-net traps than adults could be due to naïve nature of hatchlings; however, it is also possible hatchling turtles were more attracted to our baits, given that most *Pseudemys* species are thought to be omnivorous as hatchlings but predominantly herbivorous as adults (Degenhardt, Painter & Price, 1996; Fields et al., 2003; Lindeman, 2007). In general, freshwater turtle population studies yield very low, if any, hatchling captures or observations. This phenomenon could be due to naturally high mortality rates of young age classes or specific habitat and food preferences of juvenile turtles (Souza & Abe, 1998; Micheli-Campbell et al., 2013). Gibbons (1990) speculated that the survivorship of hatchling turtles is probably high once they reach the water but being more cryptic makes them difficult to study. Thus, our study system represents a potential unique opportunity to conduct multiyear sampling to shed new light on survival probabilities and somatic growth rates of hatchling freshwater turtles in relation to river hydrology and other environmental conditions. This information is necessary to effectively evaluate the tradeoffs between potential river flow management decisions for delivering water for human use and the status of *P. gorzugi* populations, especially given the hydrology of the Pecos River and its tributaries have been altered through the construction of dams and water diversions and this area is currently facing increased water demands for fracking during oil and gas extractions.

## Conclusion

We compared the efficacy of visual surveys to traditional capture–recapture surveys via baited hoop-net traps for monitoring *P. gorzugi* populations. Although we found hoop-net traps to be an effective survey method, estimating abundance from trap data required a high trap effort that may not be practical when monitoring the species at a larger spatial scale over the long term. In particular, our data thus far suggests that capture–recapture surveys require a minimum of 400 trap days per river km to obtain sufficient data to estimate abundance while accounting for imperfect detection. We found that the abundance estimates from six distance sampling surveys are highly variable but that the survey with the highest number of observed turtles was in agreement with the capture–recapture methods, albeit the estimate had larger standard errors. Given we did find some agreement in the estimates of abundance between the two survey methods and that visual surveys do not require intensive efforts in the field and are less invasive, we recommend the utility of distance visual surveys be further explored at more sites with a greater range in habitat conditions. This information is useful as planning is currently underway to expand monitoring efforts to a larger portion of the species’ range to support informed management and policy decision making by state and federal natural resource agencies.

## Supplemental Information

10.7717/peerj.4677/supp-1 Supplemental Information 1 Capture recapture survey data. Raw capture recapture data set used to derive abundance estimates of Rio Grande cooter.

10.7717/peerj.4677/supp-2 Supplemental Information 2 Visual distance sampling survey data. Raw data set of six visual distance surveys of Rio Grande cooter, including survey occasion, perpendicular distance of turtle from the observer, and class (adult or juvenile).

10.7717/peerj.4677/supp-3 Supplemental Information 3 Christman & Kamees, unpublished data. Final report submitted by Christman & Kamees to New Mexico Department of Game and Fish. Christman & Kamees studied distribution of P. gorzugi in New Mexico 2006-2007.

10.7717/peerj.4677/supp-4 Supplemental Information 4 C. W. Painter, unpublished data. Data set containing C.W. Painter’s P. gorzugi capture data in years 1992, 1993, and 2000. This data was obtained from current New Mexico state herpetologist Leland Pierce. He compiled this data from C.W. Painter field notes. Painter was a state herpetologist for NMDGF for several decades (until his death in 2015).

10.7717/peerj.4677/supp-5 Supplemental Information 5 R code used for capture-recapture and distance sampling estimates presented in the paper. Section A shows the code for the model of Huggins used to fit capture-recapture data. Model fitting used WinBUGS software 1.4 called from program R with package R2WinBUGS. Section B shows the code for multinomial-Poisson mixture model used to fit distance sampling data, using the function distsamp in the package unmarked called from program R.

## Additional Information and Declarations

### Field Study Permissions

The following information was supplied relating to field study approvals (i.e., approving body and any reference numbers): Field work was approved by New Mexico Department of Game and Fish (Authorization #3621).

### Data Availability

The following information was supplied regarding data availability: The raw data are provided in the Supplemental Files. Supplemental File 1 contains raw capture recapture data of Rio Grande cooter along the Black River, NM. The data includes individual mark, class (adult or juvenile), site (upstream or downstream), and survey results. The data was used to fit the model of Huggins and estimate the abundance while accounting for imperfect detection. Supplemental File 2 contains raw data of six Rio Grande cooter visual surveys along the Black River, NM. The data includes survey occasion (Survey No), the distance at which the turtle was observed, and class (adult or juvenile). The data was used to fit hierarchical distance sampling model and obtain turtle density while accounting for imperfect detection.
