# Supplementary material for: Comparison of hoop-net trapping and visual surveys to monitor abundance of the Rio Grande cooter (Pseudemys gorzugi)
Source: PeerJ. 2018 May 11;6:e4677. doi: 10.7717/peerj.4677 (PMC5951123; doi:10.7717/peerj.4677)
Supplement: Supplemental Information 3 — Final report submitted by Christman & Kamees to New Mexico Department of Game and Fish. Christman & Kamees studied distribution of P. gorzugi in New Mexico 2006-2007. [file peerj-06-4677-s003.pdf]

Current Distribution of the Blotched Watersnake (*Nerodia erythrogaster transversa*) and the Rio Grande Cooter (*Pseudemys gorzugi*) in the Lower Pecos River System Eddy County, New Mexico 2006–2007.

Final Report to:

Share with Wildlife Program
New Mexico Department of Game and Fish
Contract # 06-516.0000.029

Submitted by:

Bruce L. Christman
736 Cardenas SE
Albuquerque, NM 87108

and

Larry K. Kamees
8505 San Juan NE.
Albuquerque, NM 87108

June 30, 2007

Table of contents

Introduction	2
Methods	3
Results	4
Discussion	7
Recommendations	10
Acknowledgements	11
Literature Cited	12
Appendix 1: Scutellation and morphometric data for *Nerodia erythrogaster*.	14
Appendix 2: Stomach contents of *Nerodia erythrogaster transversa* from the Chihuahuan Desert Region of New Mexico, Texas, and Coahuila Mexico.	15
Appendix 3: Neonate data New Mexico	16
Appendix 4: Photos of juvenile and adult *Nerodia erythrogaster* and *Pseudemys gorzugi* Eddy Co. New Mexico.	17
Appendix 5: Photos of habitats where *Nerodia erythrogaster* and *Pseudemys gorzugi* were found, Eddy Co. New Mexico, 2006.	19
Appendix 6: Riparian obligate amphibians and reptiles encountered during 2006-2007 trapping efforts for *Nerodia erythrogaster* and *Pseudemys gorzugi*.	22

INTRODUCTION

Objectives

The primary objective of this project was to investigate the current distribution and status of the Blotched Watersnake (*Nerodia erythrogaster transversa*) and Rio Grande Cooter (*Pseudemys gorzugi*) in southeastern New Mexico. A secondary objective was to document other riparian obligate amphibians and reptiles in the lower Pecos River drainage and associated secondary drainages in southeastern New Mexico. We also conducted a food habits and reproductive investigation of wild caught specimens in New Mexico and museum specimens from the Chihuahuan Desert. The distribution of the Blotched Watersnake in New Mexico falls within the Chihuahuan Desert physiographic region (Morafka 1974) and therefore it is believed that prey species available to snakes in New Mexico would be most similar to those found elsewhere in the Chihuahuan Desert.

Blotched Watersnake (*Nerodia erythrogaster transversa*)

Nerodia erythrogaster ranges from southern Michigan and southern Delaware to the coastal states in Coahuila, and Nuevo Leon, in the south with isolated localities in Durango and Zacatecas, Mexico. From east to west it is known from the Atlantic coast to western Oklahoma, and southeastern New Mexico in the west (Conant and Collins 1991, Degenhardt et al. 1996). There are currently six recognized subspecies of Plain-bellied Watersnake (*N. erythrogaster*) with only *N. e. transversa* found in New Mexico. The range of *N. e. transversa* includes western Missouri, Kansas, northwestern Arkansas, central Texas, southeastern New Mexico and northern Coahuila, Nuevo Leon and Tamaulipus, Mexico (Conant and Collins 1991, Gibbons and Dorcas 2004). In New Mexico *N. e. transversa* exists at the western limit of its range and is known from the Lower Pecos River drainage including the Black River, Delaware River, and Rocky Arroyo in Eddy County (Degenhardt et al 1996). Currently *N. erythrogaster* is state listed as endangered by New Mexico Department of Game and Fish (NMDGF 2000). According to Gibbons and Dorcas (2004), the Natural Heritage Program rating system lists New Mexico populations as “critically imperiled – imperiled, while Oklahoma and Texas populations are listed as “apparently secure” and “secure”. Being at the western limits of its range in New Mexico, populations of *N. erythrogaster* are less dense and perhaps more likely to be fragmented through habitat loss, resulting in categorization of endangered or imperiled. Investigations conducted on *N. erythrogaster* in New Mexico have been limited to work conducted by Painter (1991, 1993) in 1991 and 1992 during which six specimens were observed on the Delaware River.

Rio Grande River Cooter (*Pseudemys gorzugi*)

Pseudemys gorzugi has a limited distribution in the Rio Grande from Brownsville to the Big Bend region, the Pecos River drainage in western Texas, and in the Pecos River and its tributaries in southeastern New Mexico. Isolated populations also occur in northeastern Coahuila, central Nuevo Leon, and northeastern Tamaulipus, Mexico (Ernst et al. 1994, Degenhardt et al. 1996). There is an unconfirmed record from Bitter Lakes

National Wildlife Refuge in Chaves County, but is otherwise currently known from below Brantley Lake downstream on the Pecos River, throughout the Black and the Delaware rivers in Eddy County, New Mexico (Degenhardt et al. 1996). An individual (C. Newsom) reported seeing *P. gorzugi* in Rocky Arroyo in 2005; this sighting was confirmed (Aug 2006) during the course of this investigation. *Pseudemys gorzugi* is currently listed as threatened by New Mexico Department of Game and Fish (NMDGF, 2006). Investigations conducted on this species have been limited to work during the field seasons of 1991–1993 conducted by C. W. Painter (NMDGF), during which time mark-recapture and radio telemetry studies were conducted at the headwaters of the Black River (C. W. Painter *unpub. data*).

METHODS

Nerodia erythrogaster transversa and other semi-aquatic snake species were captured during April–September 2006 and April–May 2007, using double-ended funnel traps set along shorelines of riverine systems with 10–20 centimeters of depth at localities of historic occurrence. Traps were generally set for 15–48 hours at a given site. Traps were checked and any snake found was transferred to a cloth snake bag for transport to a processing location. In addition, active diurnal and nocturnal searches were conducted at selected sites to hand capture any snakes observed. Data collected for snakes included weight to the nearest 0.5 gram (g.) using Pesola spring scales, and snout-vent and tail lengths to the nearest millimeter (mm). Snakes were divided into three age classes based on size (adults ≥400 mm SVL, juveniles 201–399 mm SVL, and neonates ≤ 200 mm SVL). It should be noted that these divisions are somewhat arbitrary and are not based on histological examination of gonads. Gibbons and Dorcas (2004) state that sizes of adults and juveniles vary between geographic region and within sub-species. Sexually mature females are likely larger than 400 mm SVL. Female snakes likely mature a year or so later than males. Mensural data included scale counts (ventrals, sub-caudals, mid-dorsals, and labials). All watersnakes were palped for stomach contents and a blood or tissue sample collected for future genetic analysis. All watersnakes were tagged using PIT tags (passive integrated transponders) for future individual identification and released at the point of capture.

Museum specimens of *N. e. transversa* were borrowed from nine museum collections to investigate diet. Localities were restricted to the Chihuahuan Desert region of New Mexico, Trans-Pecos Texas, and Coahuila Mexico to reflect similar habitats of species occurrence and available prey species. Snakes were measured (SVL and tail length), and mensural data taken. All snakes were then opened to examine the stomach contents and prey items were identified to the lowest taxonomic level.

Pseudemys gorzugi and other aquatic turtle species were captured using hoop traps with one funnel opening, baited with sardines, and set along the shorelines of riverine habitats with ca. ≥ 1 m of water depth. Turtle trapping was conducted during April–September 2006 and April–May 2007. Habitats with sufficient water clarity were actively searched using mask and snorkel in an effort to hand capture any turtles encountered. Observations of turtles were made at some sites using binoculars to identify

species. Turtle data collected included curved carapace length (CCL), straight-line carapace length (SCL), plastron length (PL), shell height (SH), shell width (SW) and mass (g.). All measurements were recorded in millimeters. *Pseudemys gorzugi* were separated into two age classes based on size; adult SCL ≥ 140–150 mm (males-females) and juveniles SCL ≤ 140 mm. These size classes are arbitrary as exact sizes of maturation are unknown. Trapping sites were determined from gray literature (NMDGF files), personal communications with C. W. Painter, and museum records from the Museum of Southwestern Biology (MSB) at the University of New Mexico (UNM) in Albuquerque.

RESULTS

Twenty-five sites were surveyed for *N. e. transversa* and 24 sites for *P. gorzugi* between April 10 and June 15, 2006 and April 3 and May 30, 2007 (Appendices 1, 2 & 3). A total of 28,927 and 5,094 trap hours were spent attempting to document *N. e. transversa* and *P. gorzugi*, respectively. This equates to 964 trap hours/snake and 98 trap hours/turtle. Additionally, 45 person hours were spent actively searching for *N. e. transversa* (n=11) equating to 4.1 person hours required for finding one snake. A total of 29 person hours were spent actively searching for *P. gorzugi* (n=4) equating to 7.3 person hours required to find one turtle.

Nerodia erythrogaster

We attempted to survey historic localities however, several of the locality descriptions were ambiguous and could not be positively located. *Nerodia erythrogaster transversa* were captured at 7 of 25 sites surveyed with a total of 41 captures including one recapture. The single recaptured female occurred at the old diversion dam on the Delaware River (Site 23) 24 days later and within 1 meter of the initial capture suggesting some site fidelity. The recaptured snake increased in SVL from 580 mm to 620 mm (40 mm) and increased in mass from 127.5 g. to 152.5 g. (25 g.). A single *N. e. transversa* voucher specimen was collected on the Pecos arm of Avalon Reservoir (BLC-586). Fifteen of the 40 snake captures occurred in the Pecos River between NM Hwy 31 and "10 Mile Dam". Of the 40 captures, 20 were males (16 adult, 4 juvenile); and 21 were females (15 adults, 6 juveniles). Lengths of *N. e. transversa* captured are summarized in Appendix 1. Figure 1 shows the length to mass relationship for all snakes captured illustrating the sexual dimorphism exhibited in watersnakes.
Females reach significantly larger body sizes than males in length and mass greater than 650 mm SVL.

A single observation of *N. e. transversa* was made in 2006 at the spring near the flume at the north end of Carlsbad Municipal Lake (Site 8) (M. Campbell *pers. comm.*). *Nerodia erythrogaster transversa* were not observed or trapped at Sitting Bull Falls.
In addition to *N. e. transversa*; two other snake species were encountered during surveys, *Thamnophis proximus* and *T. marcianus*. *Thamnophis proximus* were captured on the Pecos River at NM Hwy 31(N = 4), at Rocky Arroyo at NM Hwy 137 (N = 1), and observed on the Delaware River at US Hwy 285 (N = 1), and *T. marcianus* were found on the Delaware River at the old diversion dam, Pecos River above Red Bluff Reservoir, and at Sitting Bull Falls. Scutellation data of *N. erythrogaster* were collected and

compared to those reported by Degenhardt et al. (1996) and Gibbons and Dorcas (2004) (Appendix 1).

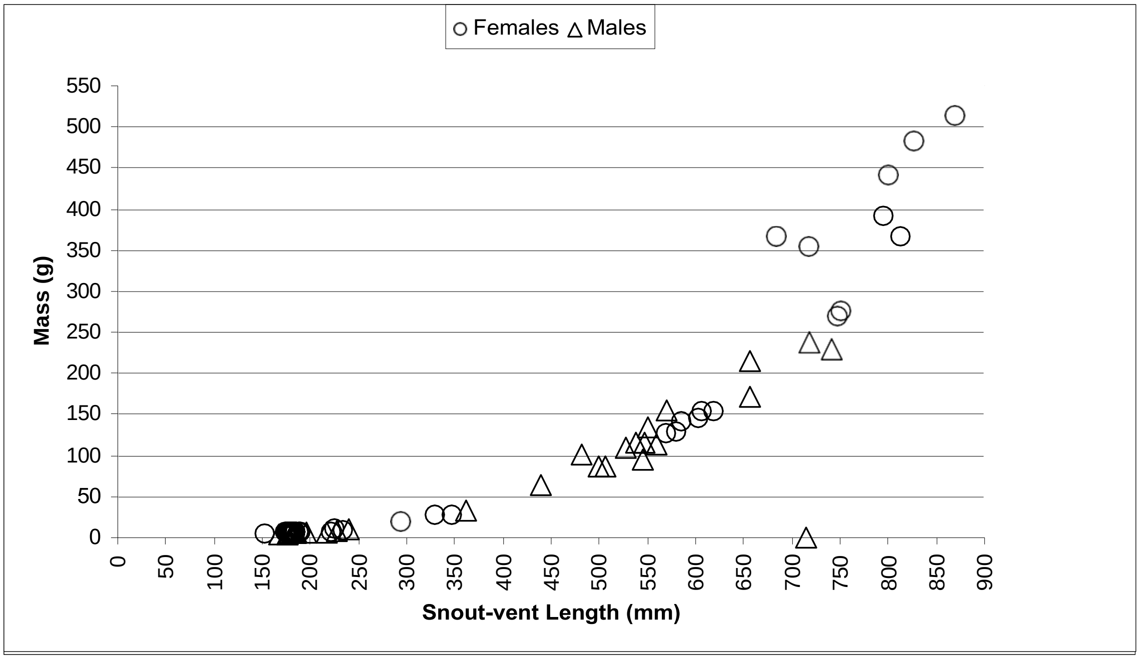

Figure 1. *Nerodia erythrogaster* length-weight relationship for Eddy County, 2006-2007.

Notes on food habits

Food habits were investigated by examining 41 wild caught specimens and 128 museum specimens (Appendix 2). Twenty-nine food items were palped from 14 of 41 total *N. e. transversa* captured, and 17 prey items were recovered from 14 museum snakes. Fish were the dominant item recovered from 13 watersnakes (8 Carp [*Cyprinus carpio*], 7 Red Shiners [*Cyprinella lutrensis*], 4 Green Sunfish [*Lepomis cyanellus*], 1 Channel Catfish [*Ictalurus punctatus*], 1 Largemouth Bass (*Micropterus salmoides*], 1 Rainwater Killifish [*Luciana parva*], 1 Rio Grande Shiner [*Notropis jemezanus*], 1 Fathead Minnow [*Pimephales promelas*], and 3 unidentified fish parts), and one Northern Cricket frog (*Acris crepitans*) was recovered from 1 watersnake. Furthermore, upon checking a trap a watersnake was observed with a *Lepomis cyanellus* in its mouth. Prey items from 14 museum specimens from the Chihuahuan Desert region examined were also mostly fish; 2 mosquito fish (*Gambusia* sp.), 2 Headwater Catfish (*Ictalurus lupus*), 1 unidentified catfish (Ictaluridae), 2 unidentified sunfish (Centrachidae), 3 unidentified minnow (Cyprinidae), with 1 adult leopard frog and 1 tadpole (*Rana berlandieri*), 2 Northern Cricket Frogs (*A. crepitans*), and 1 unidentified tadpole (Appendix 2).

Notes on reproduction

Reproduction information was gathered opportunistically (Appendix 3). Watersnakes are viviparous (live bearing). We captured two female *N. erythrogaster* 18 Aug 06 (Site 7), and 20 Aug 06 (Site 9), which were held until parturition, 23 Aug 06 (Site 7) and 31 Aug 06 (Site 9). These females gave birth to litters of 12 and 20 young

each (female/males = 8/4 and 8/12) with a combined sex ratio of 50:50. A third gravid female with 14 follicles, was captured earlier in the season (15 June 06), but was released at the time of capture. Fifteen adult females were captured, 12 of which were ≥ 600 mm SVL (minimum size of a gravid snake from our data and Conant 1969) and 3 of these were gravid. These data indicate approximately 25% of sexually mature females may be gravid/birthing in a given year in New Mexico. The data we gathered from wild snakes, museum specimens and literature (Conant 1969), indicated snakes may be detectably gravid as early as mid June. Data from nine female snakes from New Mexico and Coahuila indicate late summer into fall for dates of parturition (4 August–29 November avg. = mid. September). For New Mexico we have two wild caught snakes and a late term museum specimen all which indicate late August – mid September for likely dates of parturition. Brood sizes for New Mexico snakes examined averaged 18 (12–25), and for all Chihuahuan Desert specimens examined brood sizes averaged 9 (2–25).

Pseudemys gorzugi

Pseudemys gorzugi were found at 11 of 24 sites surveyed, with a total of 56 captures. Of the 11 sites *P. gorzugi* occur in New Mexico, they were most abundant on the Black River, particularly in the upper reaches where *P. gorzugi* were the dominant species encountered. Prior to this investigation, *P. gorzugi* had not been documented from Rocky Arroyo, however during one trapping period we captured 11 *P. gorzugi* of various size classes, and collected one voucher specimen (BLC-582). *Pseudemys gorzugi* were only found at one site on the Delaware River (old diversion dam) where three adults were trapped and a shell was recovered with an obvious bullet hole. Sites surveyed on the Pecos River had relatively few individuals encountered per trapping effort. At the Pecos River at NM Hwy 31 (Site11) a single juvenile was hand captured, at 10 Mile Dam (Site10) a single large adult female was trapped, and at the Pecos River at the Black River (Site12) confluence a single adult female was trapped. The site below Avalon Dam (Site 6) was not actively trapped however *P. gorzugi* were relatively common with various size classes observed. An observation (with photos) of two basking *P. gorzugi* was made by D. Reily at Carlsbad Municipal Lake June 2006, and we captured a single female near the flume at the upstream end of Carlsbad Municipal Lake (Site 8), verifying their continued presence at that site. Another observation was made at Rattlesnake Springs of a single adult *P. gorzugi* on 5 May 2006 (BLC *pers. obs*.). These observations were not included in data tables or maps. Photographic examples of adult and juvenile snakes are in Appendix 4, and habitats in Appendix 5. A total of 281 turtles were captured during this investigation including five additional species; Western Painted Turtle (*Chrysemys picta*) N = 7, Red-eared Slider (*Trachemys scripta*) N = 145, Common Snapping Turtle (*Chelydra serpentina*) N = 6, Spiny Softshell Turtle (*Apalone spinifera*) N = 56, and Yellow Mud Turtle (*Kinosternon flavescens*) N = 12.

In addition to other species of turtles and snakes, three amphibian species (*Acris crepitans, Rana berlandieri*, and *Rana blairi*) were encountered. *Acris crepitans* was found at all sites surveyed except the headwaters of the Black River, the Pecos River arm of Red Bluff Reservoir, W. S. Huey Wildlife Area, Sitting Bull Falls, and Chalk Bluff

Draw. *Rana berlandieri* was observed on the Pecos River at NM Hwy 31, on the Delaware River at the "old diversion dam", Rocky Arroyo at NM Hwy 137, and at Sitting Bull Falls, and *R. blairi* only at Chalk Bluff Draw.

Pseudemys gorzugi sizes were plotted (straight-line carapace length vs. mass) in Figure 2 for those turtles with complete data. Five females exceeded our 2000 g scale, these were plotted as 2000 g, and are seen as a slightly artificial grouping with regards to weight.

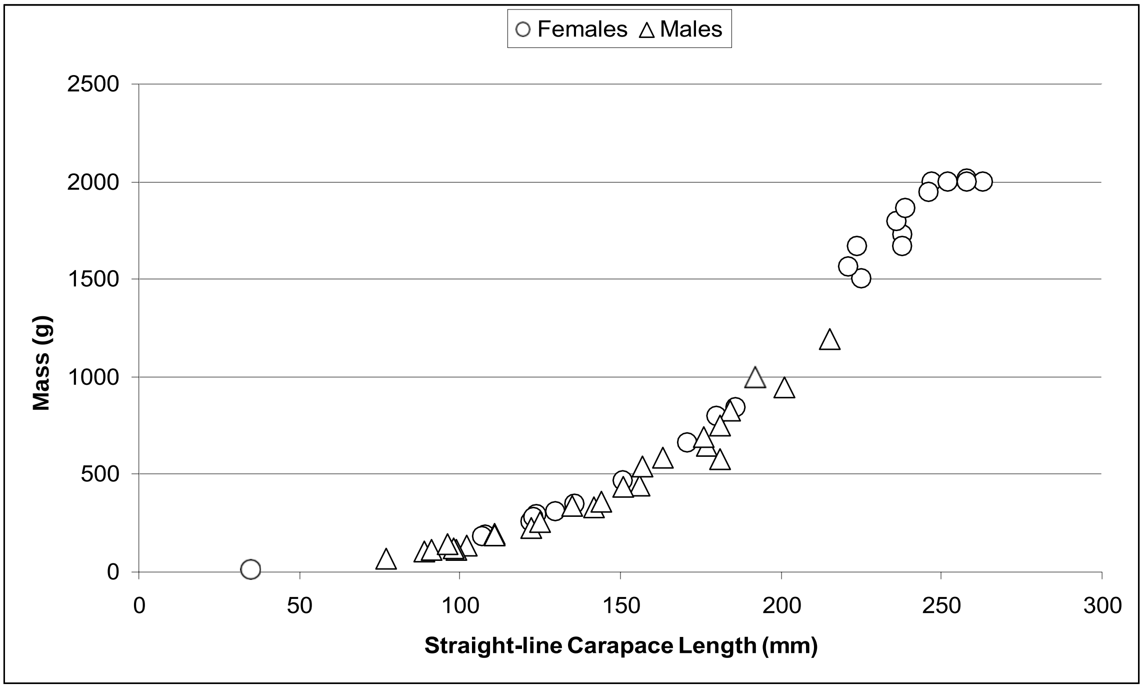

Figure 2. *Pseudemys gorzugi* length-weight relationship for Eddy County, 2006.

DISCUSSION

Nerodia erythrogaster

Distribution of *N. e. transversa* in New Mexico was not found to be substantially different than that reported in Degenhardt et al. (1996). However at several historic localities the presence of *N. e. transversa* could not be confirmed. We did not trap at Rattlesnake Springs on Carlsbad Caverns National Park, but Prival and Goode (2005) were unable to confirm their continued persistence during two seasons of amphibian and reptile surveys. W. G. Degenhardt (*pers. comm.*) reported observing an adult *N. e. transversa* at Major Johnson Springs 15 April 1978; this site is currently under water behind Brantley Dam. Major Johnson Springs represents the most upstream locality from which *N. e. transversa* have been recorded in New Mexico. Our most upstream observation of *N. e. transversa* was in the Pecos River arm of Avalon Reservoir approximately 11 river miles downstream of Major Johnson Springs.

The single recaptured female snake showed measurable signs of growth during the 24 days between captures; the change in SVL may be in part due to measuring differences (as snakes are hard to measure accurately) but the change in weight is self evident. However we lack sufficient data to attempt to estimate growth rates of *N. e. transversa.*

Regarding food habits Degenhardt et al. (1996) reported a *Lepomis cyanellus* from one trapped specimen and that snakes likely preyed on *Acris crepitans*, both of which were confirmed during this investigation. Gibbons and Dorcas reported a number of prey items for *N. erythrogaster* including; *Lepomis humilis* and various anurans, but not *L. cyanellus*, *C. lutrensis* or *A. crepitans*. These two fish species and one anuran were found to be quite common at sites surveyed during this investigation. In a previous study Mushinsky et al. (1982) showed an ontogenetic shift in diet among *N. e. flavigaster* with juveniles feeding on fish and switching to frogs as adults. This does not appear to be the case with *N. e. transversa* in NM. Our data suggests *N. e. transversa* is more opportunistic possibly due to its limited distribution and available habitat. Snakes along the Pecos River drainage and associated secondary drainages appear to prey upon species according to abundance and size. Juvenile and adult snakes alike contained a variety of prey species (Appendix 2). In the broader Chihuahuan Desert region juvenile snakes were found to take small frogs (*A. crepitans*), tadpoles, and small fish. Adult snakes were found to take fish of various sizes (were not prey size limited), with a single specimen which ate one adult leopard frog (*R. berlandieri*).

The Pecos River site upstream of NM Hwy 31 to "10 Mile Dam" (Site 11) appears to have the most robust population despite being heavily used by fishermen, weekend campers and partiers. The habitat is more diverse here than immediately up or down stream, with a divided channel over bedrock that increases water flow rate and likely oxygen content. Water depths are relatively shallow (≤ 1.5 m) and riparian obligate plant species such as sedges, cattail, and rushes are abundant creating a thick maze of potential cover and foraging habitat for *N. e. transversa* and potential anuran prey as well as other reptile species like *Thamnophis proximus*. This reach of river more than any other surveyed appears to have greater biotic diversity than was observed else where on the Pecos or its tributaries. The lack of recaptured *N. e. transversa* from this site on the Pecos River suggests a relatively robust population; however we lack data necessary to make population estimates. This site would provide an excellent study site for a radio telemetry study to investigate the habitat use of *N. e. transversa* in a habitat with heavy public use.

Preliminary observations suggest that *N. e. transversa* are less likely to be encountered in habitats with deep water (> 2 m) or at least those lacking shallows, and there seems to be some preference to moving water with rocky retreats or foraging areas. A preference for shallower water over rocky substrates may provide better foraging habitat as more fish species of appropriate size may be present in greater numbers and water clarity may be better for visual hunting.

Sexual dimorphism in *Nerodia* in general is based on size differentiation, with females larger than males. Figure 1 shows that female *N. e. transversa* weights become proportionately heavier somewhere between 650–750 mm SVL than that of males. Up to 650–750 mm SVL males and females are of a similar size. While exact age/size of

sexual maturity is unknown for *N. e. transversa*, we could not find gravid female snakes smaller than 600 mm SVL. We examined data from 49 adult females (26 museum, 7 wild caught, and 6 from literature) to access sexual maturity.

Habitat loss or degradation through human activities has been proposed as reasons for declines in the species further downstream on the Pecos River in Texas (Scudday 1974). Currently the Pecos River in New Mexico appears to be sufficiently "healthy" to support a variety of aquatic amphibians and reptiles and their prey.

Pseudemys gorzugi

Sites in need of further investigation include Brantley Lake, Carlsbad Municipal Lake, Willow Lake, and the Pecos River at Six Mile Dam. Willow Lake and Blue Spring were not investigated due to private land access constraints, but are both localities of historic occurrence. *Pseudemys gorzugi* were observed below Six Mile Dam, but were not captured.

Shooting of turtles has historically been problematic at sites along the Delaware River C. W. Painter (*pers. comm.*) found numerous turtle shells at a drying pool with bullet holes, some of which could be identified as *P. gorzugi*. Our finding of an individual *P. gorzugi* shell, at the "old diversion dam on the Delaware River, with a bullet hole indicates that turtle shooting remains a problem in this region. This type of behavior can have serious impacts on populations of such long lived animals. Recent investigations conducted on *P. gorzugi* in Texas suggest that some populations may be reduced through commercial collecting, and that New Mexico populations may be some of the most robust currently known in the wild (B. Stearns *pers. comm.*).

While both *N. erythrogaster* and *P. gorzugi* are distributed throughout the lower Pecos drainage in New Mexico, they are not evenly distributed with regard to populations. Several areas stand out as important habitats for each species. *Nerodia erythrogaster* were found to be most abundant at Rocky Arroyo, Pecos River upstream of NM Hwy 31, and on the Delaware River at the old diversion dam. Each of these sites had a variety of aquatic habitats including deep water pools, shallows, and rocky bottomed riffles and moderate to extensive riparian obligate vegetation (grasses, sedges, rushes or cattail). *Pseudemys gorzugi* were most abundant in the upper reaches of the Black River with reduced numbers encountered close to the Pecos River confluence. Rocky Arroyo contained habitat that mirrored the upper Black River and these two localities appear to have the best habitat for turtles. The best habitats seemed to be dominated by steep sided channels or pools with riparian obligate plants and a partial cottonwood (*Populus* sp.) or willow (*Salix* sp.) over story. Much of the main stem Pecos is of deeper water lacking streamside vegetation and often dominated by non-native Tamarisk (*Tamarix ramosissima*). In addition to physical habitat characteristics, water quality parameters that are altered by a regulated hydrologic regime may affect the persistence of native fauna within aquatic habitats of the lower Pecos River (Lang 2001). While we lack the data to assess habitat parameters, our observations indicate the greater diversity of natural habitats/systems the greater the occurrence of *N. erythrogaster* and *P gorzugi*. The Pecos River drainage and associated tributaries face the same challenges as most southwest river systems, including avoiding continued degradation due to ongoing

use and little reclamation. Much of the Pecos is inundated with Tamarisk, occupied by exotic fish (carp), and used as a dumping ground.

RECOMENDATIONS

Current New Mexico state listings for *N. erythrogaster* and *P. gorzugi* should maintain the status quo. Further investigations should include radio telemetry studies into habitat use by *N. erythrogaster* at sites with heavy public use. Increased education and law enforcement may help protect turtles and snake populations from further persecution.

Acknowledgements

We would like to thank J. Borgmeyer, D. Burkett, L. Burkett, C. Crowder, T. Cotten, E. Gilbert, A. Lamb, M. Martzoff, C. Painter, B. Stearns, N. Stearns, M. Reily and D. Riely for assistance with fieldwork. C. Painter and T. Nelson with site locality information and access information. H. Brandenburg and M. Farrington for identification of fish from stomach samples. L. W. Gregory for granting permission for land access.

We would like to thank the following institutions for allowing us to borrow specimens for stomach content analysis: Eastern New Mexico University, Museum of Southwest Biology University of New Mexico, Sul Ross State University, Los Angeles County Museum, Field Museum of Natural History, University of Kansas Museum of Natural History, Texas A&M University Cooperative Wildlife Collection, Texas Natural History Collection, and University of Michigan Museum of Zoology.

Literature Cited

Conant, R. 1969. A review of the water snakes of the genus *Natrix* in Mexico. Bulletin of the American Museum of Natural History 142:1–140.

Conant, R. and J. Collins. 1991. A Field Guide to Reptiles and Amphibians of Eastern and Central North America. 3rd. Edition. Houghton Mifflin, Boston.

Degenhardt, W. G., C.W. Painter, and A. H. Price. 1996. Amphibians and Reptiles of New Mexico. Univ. New Mexico Press, Albuquerque. 431 pp.

Ernst, C. H., J. E. Lovich, and R. W. Barbour. 1994. Turtles of the United States and Canada. Smithsonian Institution Press. 587 pp.

Gibbons, J. W. and M. E. Dorcas. 2004. North American Watersnakes A Natural History. Univ. of Oklahoma Press, Norman. 439 pp.

Lang, B. K. 2001.Status of the Texas Hornshell and native freshwater mussels (Uninoidea) in the Rio Grande and Pecos River of New Mexico and Texas. E-38 grant completion report to U. S. Fish and Wildlife Service, Albuquerque, New Mexico. New Mexico Dept. of Game and Fish, Santa Fe, New Mexico. 13 pp. + 6 appendices.

Morafka, D. J. 1974. Is there a Chihuahuan Desert? A quantitative evaluation through a herpetofaunal perspective. Pages 437–454 *in* R. H. Wauer and D. H. Riskind, editors. Transactions of the symposium on the biological resources of the Chihuahuan Desert region, United States and Mexico. U. S. Dept. of the Interior, Natl. Park Serv., Trans. Proc. Series (3). xxii + 658 pp.

Mushinsky, H. R., J. J. Hebrard, and D. S. Vodopich. 1982. Ontogeny of water snake foraging ecology. Ecology. 63(6):1624–1629.

New Mexico Dept. of Game and Fish. 2006. Threatened and Endangered Species of New Mexico. 2006 Biennial Review. New Mexico Dept. of Game and Fish, Santa Fe, New Mexico. ix + 122 pp.

Painter, C. W. 1991. Summary of Field Work – 1991, Blotched Watersnake (Nerodia erythrogaster transversa). Unpublished Report to USDI Bureau of Land Management, Carlsbad Resource Area.

Painter, C. W. 1991. Preliminary Investigations of the Distribution and Natural History of the Rio Grande River Cooter (*Pseudemys concinna gorzugi*) in New Mexico. Unpublished Report. New Mexico Dept. of Game and Fish to Bureau of Land Management, Carlsbad Resource District. 27 p.

Painter, C. W. 1993. Preliminary Investigations of the Distribution and Natural History of the Blotched Watersnake (Nerodia erythrogaster transversa) in New Mexico. Unpublished Report to USDI Bureau of Land Management, Carlsbad Resource Area.

Prival, D. and M. Goode. 2005. Chihuahuan Desert National Parks Reptile and Amphibian Inventory. Unpublished report to USDI National Park Service. 113 p.

Scudday, J. F. 1974. Some recent changes in the herpetofauna of the northern Chihuahuan Desert, p. 513–522. In R. H. Wauer and D. H. Riskind (eds.), Transactions of the Symposium on the Biological Resources of the Chihuahuan Desert Region, United States and Mexico. U. S. Dept. of the Interior, Natl. Park Serv., Trans. Proc. Series (3). xxii + 658 pp.

Appendix 1

Scutellation and morphometric data for *N. erythrogaster*.

Scutellation *Nerodia erythrogaster*.

	Eddy Co., NM 2006 -07	Degenhardt	Gibbons and Dorcas
Subcaudals			
Females	65–78 (68)	61–79	64–74
Males	69–81 (78)	75–87	71–84
Ventrals			
Females	134–146 (144)	n/a	141–157
Male	133–155 (143)	n/a	141–156
Combined		132–159	138–158
Middorsals			
Females	22–25 (23)		21–25
Males	23		23
Combined		23–27 (25)	19–25
Supralabials			
	7–9 (8)	7–10	8
Infralabials			
	8–13 (10)	n/a	10–11

Morphometrics *Nerodia e. transversa*, Eddy Co. 2006-07.

	Adults			Juveniles		
	n	SVL	Total Length	n	SVL	Total Length
Females	15	570-870 (705)	743-1109 (894)	22	153-347 (206)	202-458 (270)
Males	16	440-741 (578)	581-971 (776)	20	167-362 (197)	226-482 (266)

Appendix 2

Stomach contents of 28 *Nerodia erythrogaster transversa* from the Chihuahuan Desert Region of New Mexico, Texas, and Coahuila Mexico. N = number of food items; % O = percent of occurrence.

Prey Item	N	% O
FISHES		
Cyprinidae (minnows)		
Cyprinus carpio	8	17
Cyprinella lutrensis	7	15
Dionda sp.	1	2
Notropis jemezanus	1	2
Pimephales promelas	1	2
Unidentified cyprinids	3	7
Ictaluridae (catfishes)		
Ictalurus lupis	2	4
Ictalurus punctatus	1	2
Unidentified ictalurids	1	2
Fundulidae (topminnows)		
Lucania parva	1	2
Poeciliidae (mosquito fishes)		
Gambusia sp.	2	4
Centrachidae (sunfishes)		
Lepomis cyanellus	3	7
Lepomis sp.	1	2
Micropterus salmoides	2	4
Unidentified centrachids	2	4
Unidentified fishes	3	7
AMPHIBIANS		
Hylidae (tree frogs)		
Acris crepitans	3	7
Ranidae (true frogs)		
Rana berlandieri	1	2
R. berlandieri tadpole	1	2
Unidentified tadpole	1	2
Total	46	

Appendix 3 . Data on two broods of *Nerodia erythrogaster transversa* from New Mexico, 2006.

Locality of Collection	Date of Collection	Date of Birth	No. of Young	SVL (mm) of Young (Range and Average)	Total Length (mm) of Young (Range and Average)	Weight (g) of Young (Range and Average)	Length of Female (mm)		Weight of Female (g)	
							Total Length (mm)	SVL (mm)	Pre-Parturition	Post-Parturition
Rocky Arroyo, NM 137	18 August 2006	23 August 2006	12	174-195 (183)	227-262 (241.3)	5.5-6.4 (5.9)	885	685	366	232
Pecos River Below 6 Mile Dam	20 August 2006	31 August 2006	20	153-188 (183.3)	202-252 (245.1)	3.2-6.2 (5.8)	919	827	481	267

[illegible]

Appendix 4: Photos of juvenile and adult *Nerodia erythrogaster* and *Pseudemys gorzugi* Eddy Co., NM.

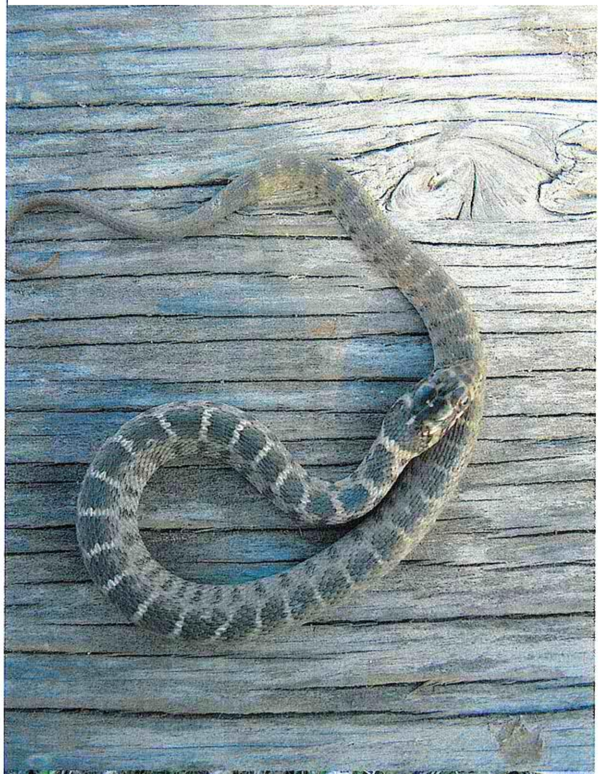

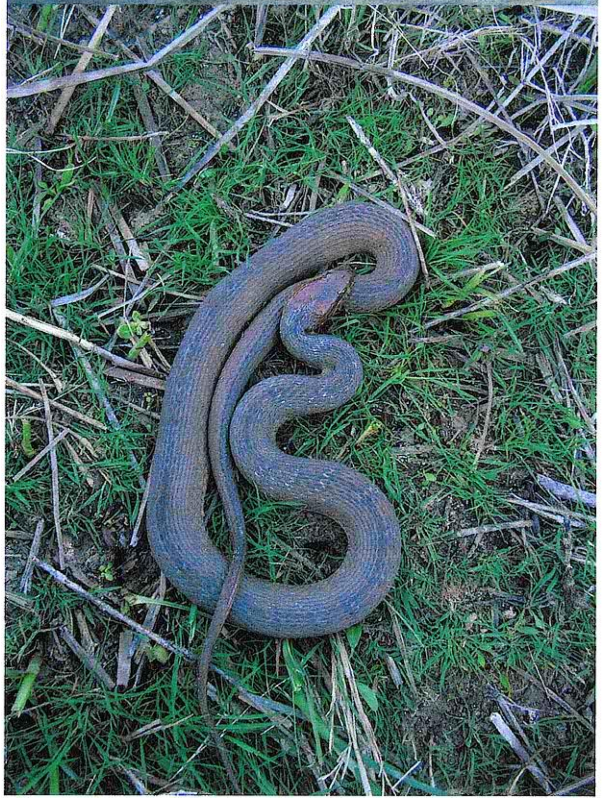

Photo 1 and 2. *Nerodia erythrogaster transversa* juvenile and adult, Pecos River.

Appendix 4 cont.

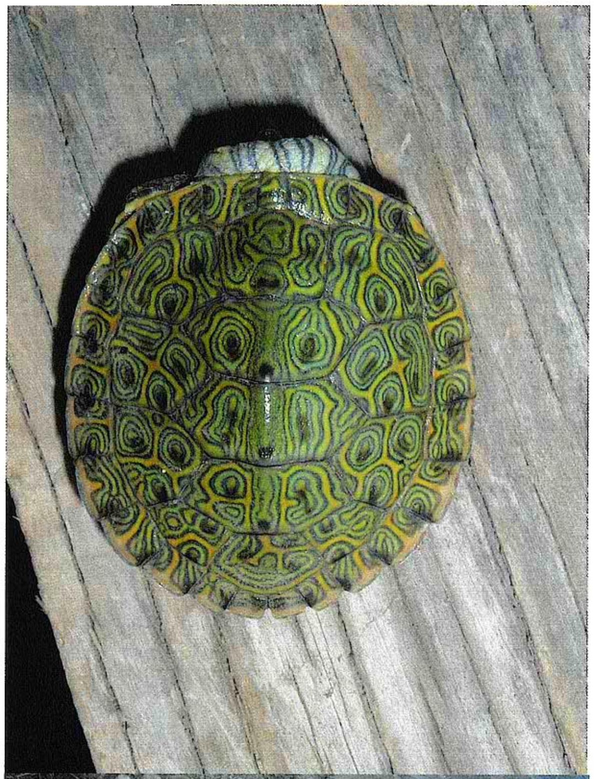

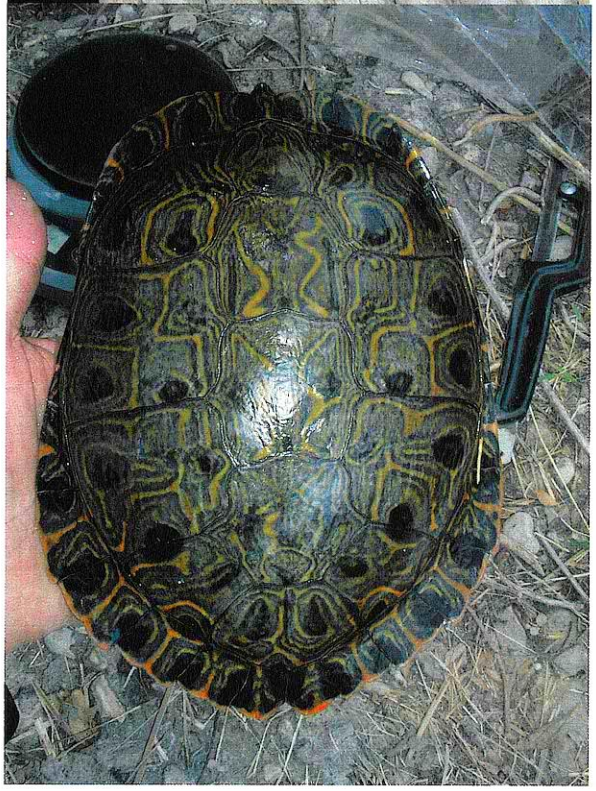

Photo 3 and 4. *Pseudemys gorzugi*, juvenile Pecos River, adult Black River.

[illegible]

Appendix 5: Photos of habitats where *Nerodia erythrogaster* and *Pseudemys gorzugi* were found, Eddy Co., NM, 2006

Habitat Photos: *Nerodia erythrogaster*

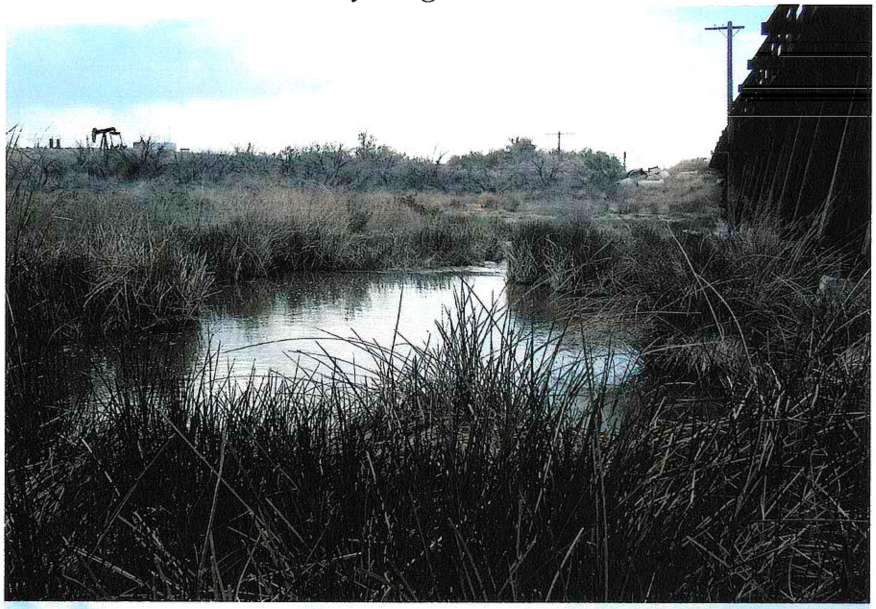

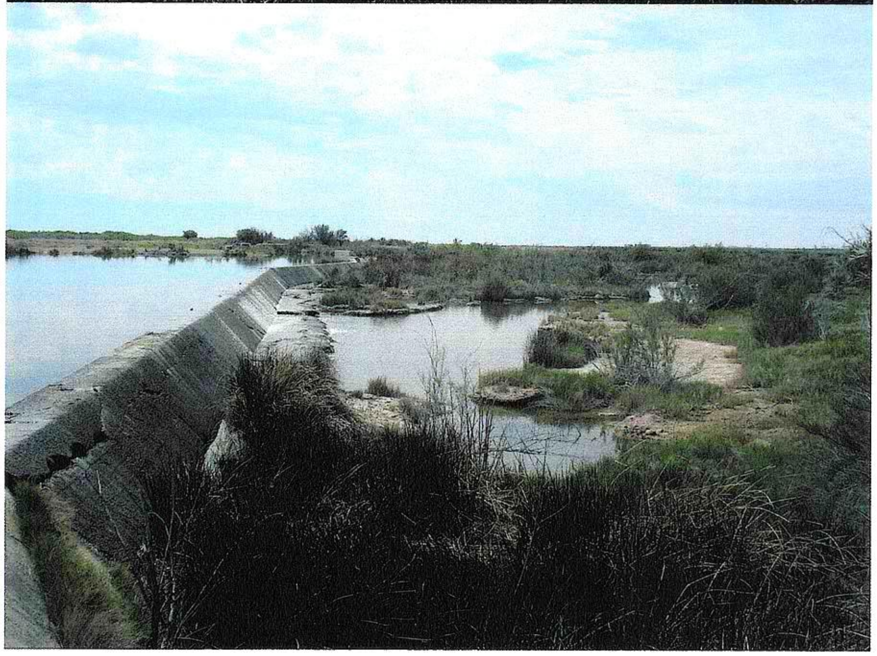

Pecos River above NM Hwy 31 (top) and at "10 Mile Dam" (bottom)

Appendix 5 cont.

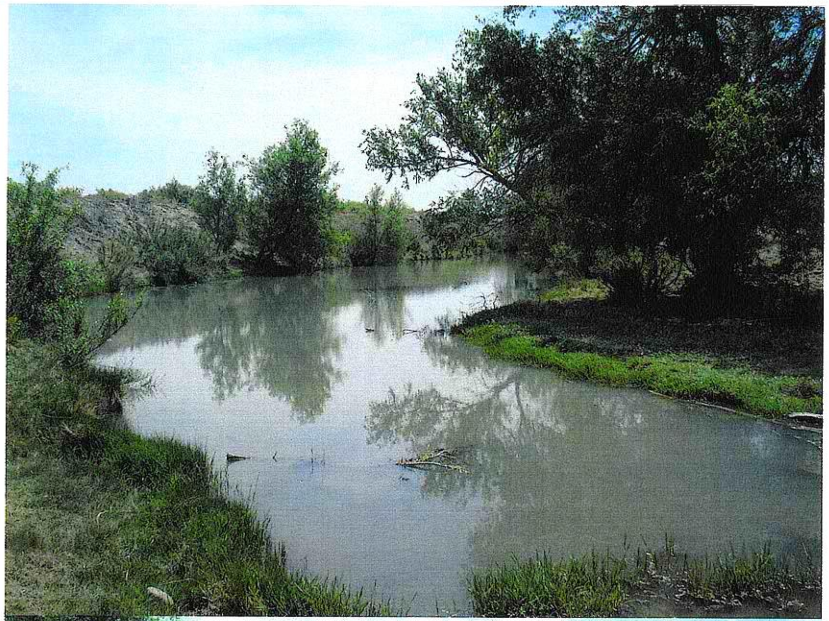

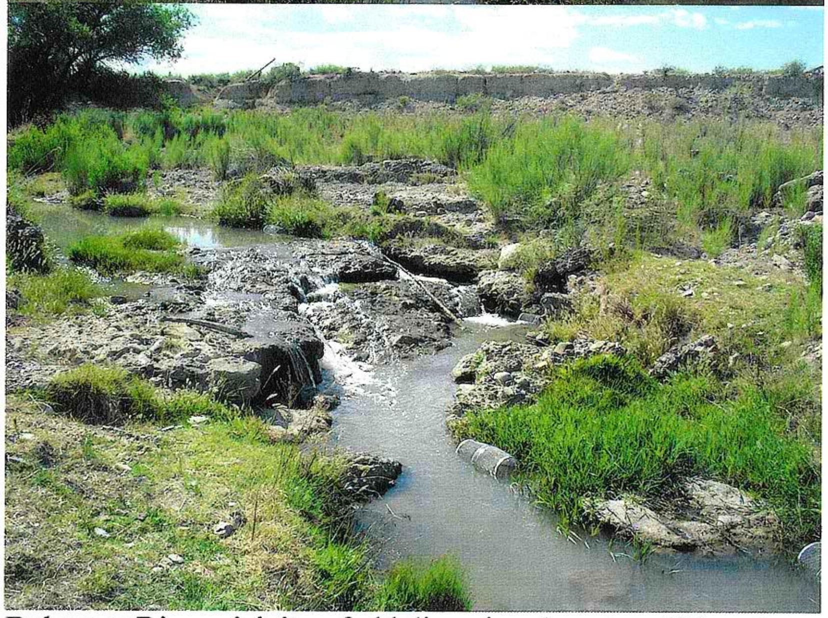

Delaware River vicinity of old diversion dam, top, (20 m above dam) pool and bottom, (100 m below dam) showing ledge habitat favored by *N. erythrogaster*.

Appendix 5 cont.

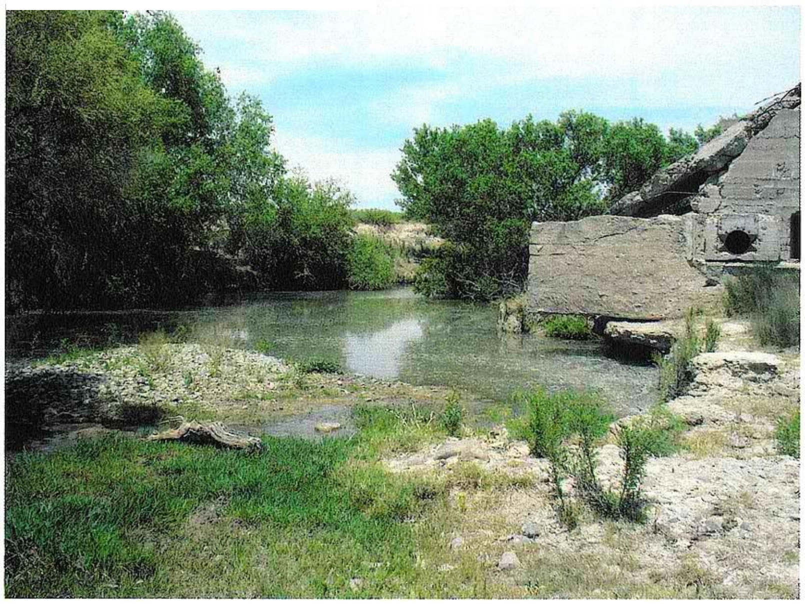

Delaware River at old diversion dam, *N. erythrogaster* and *P. gorzugi* pool habitat.

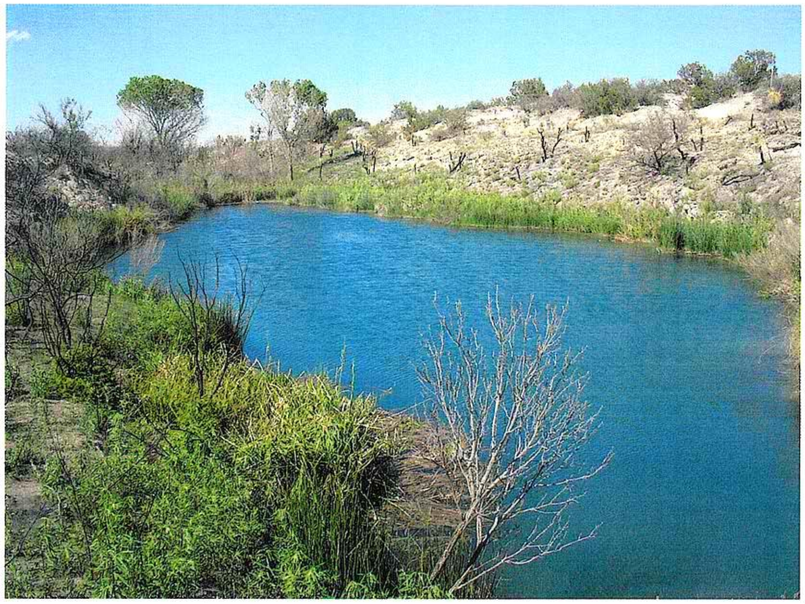

Black River, headwaters, *P. gorzugi* pool habitat.

Appendix 6

Riparian obligate amphibians and reptiles encountered during 2006 -2007 trapping efforts for *Nerodia erythrogaster* and *Pseudemys gorzugi*.

Species	Trapping locality sites
Northern Cricket Frog (*Acris crepitans*)	6, 8, 9, 10, 11, 12, 23, 24
Plains Leopard Frog (*Rana blairi*)	2
Rio Grande Leopard Frog (*Rana berlandieri*)	7, 11, 13, 23
Checkered Gartersnake (*Thamnophis marcianus*)	2, 13, 23, 24
Ribbon Snake (*Thamnophis proximus*)	7, 11, 22
Western Painted Turtle (*Chrysemys picta*)	2, 7
Yellow Mud Turtle (*Kinosternon flavescens*)	2, 6, 12
Common Snapping Turtle (*Chelydra serpentina*)	6, 7, 10, 18
Spiny Softshell Turtle (*Apalone spinifera*)	1, 3, 5, 6, 7, 10, 11, 12, 20, 21, 22, 23, 25
Red-eared Slider (*Trachemys scripta*)	1, 2, 5, 6, 7, 8, 9, 10, 11, 12, 14, 17, 18, 20, 21, 22, 23, 24, 25
