# Supplementary material for: Comparison of hoop-net trapping and visual surveys to monitor abundance of the Rio Grande cooter (Pseudemys gorzugi)
Source: PeerJ. 2018 May 11;6:e4677. doi: 10.7717/peerj.4677 (PMC5951123; doi:10.7717/peerj.4677)
Supplement: Supplemental Information 5 — Section A shows the code for the model of Huggins used to fit capture-recapture data. Model fitting used WinBUGS software 1.4 called from program R with package R2WinBUGS. Section B shows the code for multinomial-Poisson mixture model used to fit distance sampling data, using the function distsamp in the package unmarked called from program R. [file peerj-06-4677-s005.pdf]

A. R code used for estimating abundance of *Pseudemys gorzugi* using capture-recapture data

```
#Organize data
captures=matrix(c(0,0,1,0,0,0,
	0,0,1,0,0,0,
	0,0,1,0,0,0,
	0,0,1,0,0,0,
	0,0,1,0,0,0,
	0,0,1,0,0,0,
	0,0,0,1,0,0,
	0,0,0,1,0,0,
	0,0,0,1,0,0,
	0,0,0,1,0,0,
	0,0,0,1,0,0,
	0,0,0,1,0,0,
	0,0,0,1,0,0,
	0,0,0,1,0,0,
	0,0,0,1,0,0,
	0,0,0,0,1,0,
	0,0,0,0,1,0,
	0,0,0,0,1,0,
	0,0,0,0,1,0,
	0,0,0,0,1,0,
	0,0,0,0,1,0,
	0,0,0,0,1,0,
	0,0,0,0,1,0,
	0,0,0,0,0,1,
	0,0,0,0,0,1,
	0,0,0,0,0,1,
	0,0,0,0,0,1,
	0,0,0,0,0,1,
	1,0,0,0,0,0,
	1,0,0,0,0,0,
	0,1,0,0,0,0,
	0,1,0,0,0,0,
	0,0,1,0,0,0,
	0,0,1,0,0,0,
	0,0,1,1,0,0,
	0,0,1,0,0,0,
	0,0,1,0,0,0,
	0,0,1,0,0,0,
	0,0,0,0,0,1,
	0,0,0,0,0,1,
	0,0,0,0,0,1,
	1,0,0,0,0,0,
	0,1,0,0,0,0,
	0,1,0,0,0,0,
	0,1,0,1,1,0,
```

0,1,0,0,0,0,
0,0,1,0,0,0,
0,0,1,0,0,0,
0,0,1,0,1,0,
0,0,1,0,0,0,
0,0,1,0,0,0,
0,0,1,0,0,0,
0,0,1,1,0,0,
0,0,1,0,0,0,
0,0,0,1,0,0,
0,0,0,0,1,0,
0,0,0,0,1,0,
0,0,0,0,1,0,
0,0,0,0,0,1,
0,0,1,0,0,1,
0,0,0,0,0,1,
1,0,0,0,0,0,
1,0,0,0,0,0,
1,0,0,0,0,0,
0,1,0,0,0,0,
1,0,0,0,0,0,
0,1,0,0,0,0,
0,1,0,0,0,0,
0,0,0,1,0,0,
0,0,0,0,1,0,
0,0,0,0,1,0,
0,0,0,0,1,0,
0,0,0,0,1,0,
0,0,0,0,1,0,
0,0,0,0,0,1,
1,0,0,0,0,0,
1,0,0,0,0,0,
1,0,1,0,0,0,
1,0,0,0,0,0,
1,0,0,1,0,0,
1,0,0,0,0,0,
1,0,0,0,0,0,
1,0,0,1,0,0,
1,0,1,0,0,0,
0,1,0,0,0,0,
0,1,0,1,0,1,
0,1,0,0,0,0,
0,1,1,0,0,0,
0,0,1,0,0,0,
0,0,1,0,0,0,
0,0,0,1,0,0,
0,0,0,1,0,0,
0,0,0,0,0,1,

0,0,0,0,1,0,
0,0,0,0,1,1,
1,0,0,0,0,0,
0,0,1,0,0,0,
0,0,1,0,0,0,
0,0,0,0,1,0,
0,0,0,0,0,1,
0,1,0,0,0,0,
0,1,0,0,0,1,
0,0,0,1,0,0,
0,0,0,1,0,0,
0,0,0,1,0,0,
0,0,0,0,1,0,
0,0,0,0,1,0,
0,0,0,0,0,1,
0,0,0,0,0,1,
0,0,0,0,0,1,
0,0,0,0,0,1,
1,0,0,0,0,0,
0,0,1,0,0,0,
1,0,0,0,0,0,
1,0,0,0,1,0,
1,0,0,1,0,0,
0,1,0,0,0,0,
0,1,0,0,0,0,
0,1,0,0,0,0,
1,0,0,1,0,1,
0,0,0,1,1,1,
0,0,0,0,1,0,
0,0,0,0,0,1,
1,0,1,0,0,1,
1,0,0,0,0,0,
0,1,0,0,0,0,
0,0,1,1,0,0,
0,0,0,1,0,0,
0,0,0,0,0,1,
1,0,1,0,0,0,
1,0,0,0,0,0,
1,1,0,0,0,0,
1,0,0,0,0,1,
1,0,0,0,0,0,
1,0,1,1,0,0,
1,0,0,0,0,0,
1,0,0,1,0,0,
0,1,0,1,1,0,
0,0,1,0,0,0,
0,0,0,1,0,0,
0,0,0,1,0,0,

```
0,0,0,0,1,0,
0,0,0,0,1,0,
1,1,0,0,0,0,
0,1,0,0,0,0,
0,1,0,0,0,0,
1,0,1,0,0,0,
0,0,1,0,0,0,
0,1,0,0,0,0,
0,0,1,0,0,0,
0,0,0,0,0,1,
1,0,0,0,0,0,
1,0,0,0,0,0,
1,0,0,0,0,0,
1,0,0,0,0,0,
0,0,1,0,0,0,
0,1,0,0,0,0,
0,1,0,0,0,0,
0,0,1,0,0,0,
0,1,0,0,0,0,
0,0,0,1,0,0,
0,1,0,0,0,0,
0,0,1,0,0,0,
0,0,1,0,0,0,
0,1,0,0,0,0,
0,0,0,0,1,0,
0,0,0,0,1,0,
0,0,0,0,1,0,
0,0,1,0,0,0,
1,0,0,0,0,0,
0,0,0,0,0,1,
0,0,1,0,0,0,
0,0,1,0,0,0,
0,0,1,0,0,0,
0,0,1,0,0,0,
0,0,0,0,1,0,
0,1,1,0,0,0,
1,0,0,0,0,0,
0,0,1,0,0,0),ncol=6,byrow=T)

Individual=c("2","3","4","5","6","7","8","9","10","11","12","13","14","15","16","18","19","20","21","23",
"24","25","26","29","30","31","32","33","34",
"35","36","37","38","39","40","41","42","43","44","45","46","51","55","56","57","58","61","62","63","6
6","67","68","69","70","71","72",
"73","74","75","76","77","78","79","80","81","82","83","84","86","90","91","92","93","94","96","97","9
8","99","100","101","102","103",
"104","105","106","107","108","109","112","113","116","117","118","1002","7018","100D","1949H","1
960H","1975H","1997H","2004H","2007H",
```

```
"2018H","2019H","2020H","2021H","2022H","2030H","2031H","2033H","2043H","2057H","2057H","20
58H","2059H","2060H","2069H","2073H","2074H",
"2096H","2114H","2115H","2123H","2146H","2155H","2159H","2176H","2187H","2212H","2221H","22
23H","2227H","2228H","2229H","2233H","2234H",
"2235H","2237H","2258H","2277H","2280H","2288H","2290H","83c0325","83c0335","83c0336","83c03
40","83c0341","83c034c","83c0356","83c0358",
"83c0365","83c036a","83c0372","83c037b","83c339a","83c33b3","83c33bf","83c33D4","852ee23","852
ee28","852ee38","852ee3a","852ee3e",
"852ee3f","852ee40","852ee4a","852ee51","852ee55","852ee56","852ee5c","852ee68","852ee6a","85
2ee73","852ee74","852ee79","852ee7b",
        "852ee7d","852ee83")
Stage=c("female","female","male","male","male","male","male","male","male","male","female","male"
,"male","male","Juvenile","female","female","female",
"female","female","female","male","Juvenile","Juvenile","male","male","female","male","male","femal
e","male","female","female","female","male",
"male","male","male","female","male","male","male","male","male","male","female","female","female
","female","male","male","female","male",
"female","female","male","male","female","female","female","female","male","female","female","male
","male","male","female","male","male",
"male","male","female","female","male","female","female","female","female","male","female","female
","male","male","male","male","female",
"male","male","female","male","female","male","female","male","female","hatchling","hatchling","hatc
hling","hatchling","hatchling",
"hatchling","hatchling","hatchling","hatchling","hatchling","hatchling","hatchling","hatchling","hatchling
","hatchling","hatchling",
"hatchling","hatchling","hatchling","hatchling","hatchling","hatchling","hatchling","hatchling","hatchling
","hatchling","hatchling",
"hatchling","hatchling","hatchling","hatchling","hatchling","hatchling","hatchling","hatchling","hatchling
","hatchling","hatchling",
"hatchling","hatchling","hatchling","hatchling","hatchling","hatchling","hatchling","hatchling","hatchling
","Juvenile","Juvenile",
"Juvenile","Juvenile","Juvenile","Juvenile","Juvenile","Juvenile","Juvenile","Juvenile","Juvenile","male","
Juvenile","male","Juvenile",
"Juvenile","Juvenile","female","Juvenile","male","female","male","male","male","male","female","male
","Juvenile","female","female","male",
      "female","Juvenile","male","male","male")
AH=c("A","A","A","A","A","A","A","A","A","A","A","A","A","A","A","A","A","A","A","A","A","A","A","A","A
","A","A","A","A","A","A","A","A","A","A",
"A","A","A","A","A","A","A","A","A","A","A","A","A","A","A","A","A","A","A","A","A","A","A","A","A","A",
"A","A","A","A","A","A","A","A","A",
"A","A","A","A","A","A","A","A","A","A","A","A","A","A","A","A","A","A","A","A","A","A","A","A","A","A",
"H","H","H","H","H","H","H","H","H",
"H","H","H","H","H","H","H","H","H","H","H","H","H","H","H","H","H","H","H","H","H","H","H","H","H","
H","H","H","H","H","H","H","H","H","H",
"H","H","H","A","A","A","A","A","A","A","A","A","A","A","A","A","A","A","A","A","A","A","A","A","A","A",
"A","A","A","A","A","A","A","A","A",
    "A","A","A","A")
```

```
Site=c("BLM1","BLM1","BLM1","BLM1","BLM1","BLM1","BLM1","BLM1","BLM1","BLM1","BLM1","BLM
1","BLM1","BLM1","BLM1","BLM1","BLM1","BLM1","BLM1","BLM1",
"BLM1","BLM1","BLM1","BLM1","BLM1","BLM1","BLM1","BLM1","BLM2","BLM2","BLM2","BLM2","BL
M2","BLM2","BLM2","BLM2","BLM2","BLM2","BLM2","BLM2",
"BLM2","JD","JD","JD","JD","JD","JD","JD","JD","JD","JD","JD","JD","JD","JD","JD","JD","JD","JD","JD","JD
","L1","L1","L1","L1","JD","L1",
"L1","L1","L1","L1","L1","L1","L1","L1","L2","L2","L2","L2","L2","L2","L2","L2","L2","L2","L2","L2","L2","L
2","L2","L2","L2","L2","BLM1",
"BLM1","BLM2","BLM1","BLM1","BLM1","BLM1","BLM2","BLM2","BLM2","BLM2","BLM2","BLM2","BL
M2","BLM2","BLM2","BLM2","BLM2","JD","L1","JD","JD",
"JD","JD","JD","JD","JD","JD","JD","JD","L1","L1","L1","L1","L1","L1","L2","L2","L2","L2","L2","L2","L2","L
2","L2","L2","L2","L2","L2","L2",
"L1","JD","L1","JD","JD","JD","JD","BLM1","L1","JD","L1","JD","L1","L1","L1","L1","L2","L2","L2","L2","L1
","L2","L1","L1","L1","L1","L2","L1",
      "L1","L1","L1","L2","L2","L2","L2","L1")
Loc=c("UpDistance","UpDistance","UpDistance","UpDistance","UpDistance","UpDistance","UpDistance"
,"UpDistance","UpDistance","UpDistance","UpDistance",
"UpDistance","UpDistance","UpDistance","UpDistance","UpDistance","UpDistance","UpDistance","UpDi
stance","UpDistance","UpDistance","UpDistance",
"UpDistance","UpDistance","UpDistance","UpDistance","UpDistance","UpDistance","UpDistance","UpDi
stance","UpDistance","UpDistance","UpDistance",
"UpDistance","UpDistance","UpDistance","UpDistance","UpDistance","UpDistance","UpDistance","UpDi
stance","Down","Down","Down","Down","Down",
"Down","Down","Down","Down","Down","Down","Down","Down","Down","Down","Down","Down","D
own","Down","Down","Down","Down","Down","Down","Down",
"Down","Down","Down","Down","Down","Down","Down","Down","Down","Down","Down","Down","D
own","Down","Down","Down","Down","Down","Down","Down",
"Down","Down","Down","Down","Down","Down","Down","UpDistance","UpDistance","UpDistance","U
pDistance","UpDistance","UpDistance","UpDistance",
"UpDistance","UpDistance","UpDistance","UpDistance","UpDistance","UpDistance","UpDistance","UpDi
stance","UpDistance","UpDistance","UpDistance",
"Down","Down","Down","Down","Down","Down","Down","Down","Down","Down","Down","Down","D
own","Down","Down","Down","Down","Down","Down","Down",
"Down","Down","Down","Down","Down","Down","Down","Down","Down","Down","Down","Down","D
own","Down","Down","Down","Down","Down","Down",
"UpDistance","Down","Down","Down","Down","Down","Down","Down","Down","Down","Down","Dow
n","Down","Down","Down","Down","Down","Down","Down",
     "Down","Down","Down","Down","Down","Down","Down","Down","Down","Down")
first=cbind(Individual,Stage,AH,Loc)
data=data.frame(first,captures)
colnames(data) <- c("Individual","Stage","AH","Loc","Day1","Day2","Day3","Day4","Day5","Day6")
data
table(Stage)

#subset and format data
females=subset(data,Stage=="female")
hatchlings=subset(data,Stage=="hatchling")
```

```
juveniles=subset(data,Stage=="Juvenile")
males=subset(data,Stage=="male")

#upstream (site 1) vs. downstream (site 2) and analysis of hatchlings and adults separately
# Specify model in BUGS language
sink("Huggins.BUGS")
cat("
   model{  ### capture probability model parameters
   for (i in 1:2)
   {
   beta[i]~dnorm(0,0.37)
   }
   betaOffset~dnorm(0,0.37)

   #multinomial likelihood for animals captured
   for (i in 1: nhist)
   {
   for (j in 1:occas)
   {
   ### log odds capture model
   logit(cap.pzUP[i,j])<- beta[1] + beta[2]*recap[i,j] + betaOffset
   cap.pUP[i,j]<- min(0.999,max(0.001,cap.pzUP[i,j]))
   cUP[i,j]<- pow(cap.pUP[i,j],X[i,j])*pow(1-cap.pUP[i,j],1-X[i,j])
   logit(cap.pzDOWN[i,j])<- beta[1] + beta[2]*recap[i,j]
   cap.pDOWN[i,j]<- min(0.999,max(0.001,cap.pzDOWN[i,j]))
   cDOWN[i,j]<- pow(cap.pDOWN[i,j],X[i,j])*pow(1-cap.pDOWN[i,j],1-X[i,j])

   }
   etaUP[i]<-prod(cUP[i,])
   etaDOWN[i]<-prod(cDOWN[i,])
   }
   totUP<-sum(etaUP[])
   totDOWN<-sum(etaDOWN[])

   for (i in 1:nhist) {xiUP[i]<-etaUP[i]/totUP}
   for (i in 1:nhist) {xiDOWN[i]<-etaDOWN[i]/totDOWN}

   ## number marked individuals
   udotUP<-sum(fUP[])
   fUP[1:nhist]~dmulti(xiUP[1:nhist],udotUP)

   udotDOWN<-sum(fDOWN[])
   fDOWN[1:nhist]~dmulti(xiDOWN[1:nhist],udotDOWN)

   # estimate probability first capture by occasion
   for (z in 1:occas){
   logit(pUP[z])<-beta[1]
   p00.unctUP[z] <- (1-pUP[z])
```

```
    p00UP[z] <- cut(p00.unctUP[z])
    }

    # estimate probability first capture by occasion
    for (z in 1:occas){
    logit(pDOWN[z])<-beta[1]
    p00.unctDOWN[z] <- (1-pDOWN[z])
    p00DOWN[z] <- cut(p00.unctDOWN[z])
    }

    #probability that animal is caught before end of study
    pi0UP<-1-prod(p00UP[])
    #prior on abundance
    lambdaUP~dunif(0,10000)
    ## notice the mininum is the number caught and marked
    NUP<-round(lambdaUP+udotUP)
    #binomial likelhood for abundance
    udotUP~dbin(pi0UP,NUP)

    #derived parameters
    logit(captureProbUP)<-beta[1]+betaOffset
    logit(recaptureProbUP)<-beta[1]+beta[2]+betaOffset

    #probability that animal is caught before end of study
    pi0DOWN<-1-prod(p00DOWN[])
    #prior on abundance
    lambdaDOWN~dunif(0,10000)
    ## notice the mininum is the number caught and marked
    NDOWN<-round(lambdaDOWN+udotDOWN)
    #binomial likelhood for abundance
    udotDOWN~dbin(pi0DOWN,NDOWN)

    #derived parameters
    logit(captureProbDOWN)<-beta[1]
    logit(recaptureProbDOWN)<-beta[1]+beta[2]

    Ntot<-NUP+NDOWN
    }    ",fill = TRUE)
sink()

#model setup
inits<-NULL
params<-
c("Ntot","NUP","captureProbUP","recaptureProbUP","NDOWN","captureProbDOWN","recaptureProbD
OWN","beta","betaOffset")
niter=250000
nburn=125000
nthin=1
```

```
nchains=3

#Hatchling analysis
# BUGSdata<-
list(X=Cap,nhist=nrow(Cap),occas=ncol(Cap),recap=Recap,fUP=fHatchlingsUp,fDOWN=fHatchlingsDown)
# ZZ1<-bugs(data=BUGSdata,inits=inits,parameters=params,
#         model="Huggins.BUGS",n.chains=nchains,n.thin=nthin,n.iter=niter,n.burnin=nburn,debug=F,
#         bugs.directory="C:/Users/duartead/Documents/WinBUGS14")
# print(ZZ1,digits=3)
# > print(ZZ1,digits=3)
# Inference for Bugs model at "Huggins.BUGS", fit using WinBUGS,
# 3 chains, each with 250000 iterations (first 125000 discarded)
# n.sims = 375000 iterations saved
#                   mean    sd   2.5%    25%    50%    75%  97.5%  Rhat  n.eff
# Ntot              54.318 8.151 48.000 50.000 52.000 56.000 74.000 1.001  18000
# NUP               17.471 2.998 15.000 16.000 17.000 18.000 25.000 1.001  24000
# captureProbUP      0.069 0.044  0.011  0.036  0.060  0.093  0.177 1.001 130000
# recaptureProbUP    0.047 0.034  0.007  0.023  0.039  0.062  0.136 1.001  52000
# NDOWN             36.847 5.772 32.000 34.000 35.000 38.000 51.000 1.001  20000
# captureProbDOWN    0.330 0.079  0.167  0.278  0.333  0.386  0.478 1.001  15000
# recaptureProbDOWN  0.240 0.053  0.146  0.203  0.237  0.274  0.351 1.001  95000
# beta[1]           -0.733 0.387 -1.608 -0.953 -0.695 -0.466 -0.087 1.001  14000
# beta[2]           -0.441 0.413 -1.213 -0.720 -0.454 -0.183  0.420 1.001  10000
# betaOffset        -2.084 0.777 -3.743 -2.565 -2.038 -1.546 -0.686 1.001  97000
# deviance          87.460 4.712 81.980 84.340 86.480 89.420 98.230 1.001  23000
#
# For each parameter, n.eff is a crude measure of effective sample size,
# and Rhat is the potential scale reduction factor (at convergence, Rhat=1).
#
# DIC info (using the rule, pD = Dbar-Dhat)
# pD = 2.3 and DIC = 89.8
# DIC is an estimate of expected predictive error (lower deviance is better).

#Adult analysis
BUGSdata<-
list(X=Cap,nhist=nrow(Cap),occas=ncol(Cap),recap=Recap,fUP=fAdultUp,fDOWN=fAdultDown)
ZZ2<-bugs(data=BUGSdata,inits=inits,parameters=params,
        model="Huggins.BUGS",n.chains=nchains,n.thin=nthin,n.iter=niter,n.burnin=nburn,debug=F,
        bugs.directory="C:/Users/duartead/Documents/WinBUGS14")
print(ZZ2,digits=3)
# > print(ZZ2,digits=3)
# Inference for Bugs model at "Huggins.BUGS", fit using WinBUGS,
# 3 chains, each with 250000 iterations (first 125000 discarded)
# n.sims = 375000 iterations saved
#                   mean     sd    2.5%     25%     50%     75%   97.5%  Rhat  n.eff
# Ntot              194.414 58.734 148.000 166.000 181.000 206.000 317.000 1.001  15000
# NUP                66.414 20.759  49.000  56.000  62.000  71.000 110.000 1.001  15000
# captureProbUP       0.048  0.026   0.011   0.028   0.044   0.063   0.112 1.001 100000
```

```
# recaptureProbUP     0.019  0.012   0.004   0.010   0.016   0.024   0.048 1.001 380000
# NDOWN            127.999 39.778  96.000 108.000 119.000 136.000 211.000 1.001  18000
# captureProbDOWN     0.197  0.053   0.090   0.162   0.198   0.234   0.298 1.001  16000
# recaptureProbDOWN   0.082  0.020   0.048   0.068   0.080   0.094   0.124 1.001  70000
# beta[1]          -1.444  0.375  -2.312  -1.646  -1.396  -1.186  -0.856 1.001  17000
# beta[2]          -1.004  0.424  -1.769  -1.288  -1.027  -0.749  -0.101 1.001  36000
# betaOffset        -1.700  0.647  -3.080  -2.102  -1.661  -1.253  -0.543 1.001 380000
# deviance         123.679  7.337 117.900 120.000 122.100 125.200 138.700 1.001  42000
#
# For each parameter, n.eff is a crude measure of effective sample size,
# and Rhat is the potential scale reduction factor (at convergence, Rhat=1).
#
# DIC info (using the rule, pD = Dbar-Dhat)
# pD = 4.3 and DIC = 127.9
# DIC is an estimate of expected predictive error (lower deviance is better).
#
```

B. R code used for estimating density of *Pseudemys gorzugi* using visual distance sampling

The following example is for the fifth visual survey of adult turtles

```
#Organize the data
DisFifth<-read.csv("C:\\Users\\Ivana\\Documents\\Distance In R gorzugi\\Adults Distance\\FifthSurvey.csv",header=T,sep=",")
> DisFifth
  transect distance
1        e       1
2        e       1
3        e       1
4        e       2
5        e       2
6        e       2
7        e       2
8        e       3
9        e       3
10       e       3
11       e       3
12       e       3
13       e       3
14       e       3
15       e       4
16       e       4
17       e       4
18       e       5
19       e       5
20       e       5
21       e       5
22       e       5
```

```
23      e       5
24      e       5
25      e       5
26      e       5
27      e       6
28      e       6
29      e       6
30      e       6
31      e       6
32      e       7
33      e       7
34      e       7
35      e       7
36      e       7
37      e       9
> DisFifthFormat<-
formatDistData(DisFifth,distCol="distance",transectNameCol="transect",dist.breaks=c(0,2,4,6,8,10))
> DisFifthFormat
  [0,2] (2,4] (4,6] (6,8] (8,10]
e    7    10    14     5      1
> DisFifthFrame<-
unmarkedFrameDS(y=as.matrix(DisFifthFormat),survey="line",dist.breaks=c(0,2,4,6,8,10),tlength=rep(15
00,1),unitsIn="m")
> DisFifthFrame
Data frame representation of unmarkedFrame object.
  y.1 y.2 y.3 y.4 y.5
e   7  10  14   5   1

#Running the model
> hn_Fifth_Null<-distsamp(~1~1,DisFifthFrame,keyfun="halfnorm",output="density",unitsOut="ha")
> hn_Fifth_Null
Call:
distsamp(formula = ~1 ~ 1, data = DisFifthFrame, keyfun = "halfnorm",
    output = "density", unitsOut = "ha")

Density:
 Estimate    SE    z  P(>|z|)
     2.97 0.221 13.4 3.67e-41

Detection:
 Estimate    SE    z  P(>|z|)
     1.68 0.206 8.17 2.99e-16

AIC: 29.94842

#Backtransforming the density estimates and deriving abundance estimates
>  backTransform(hn_Fifth_Null,type="state")
Backtransformed linear combination(s) of Density estimate(s)
```

```
 Estimate   SE LinComb (Intercept)
     19.6 4.33    2.97           1

Transformation: exp
> plotArea.inHectares<-1500*20/10000
> hnFifth.density<-predict(hn_Fifth_Null,type="state")$Predicted
> hnFifth.abundance<-hnFifth.density*plotArea.inHectares
(FifthN.hat<-sum(hnFifth.abundance)) #abundance estimate
[1] 58.68755
> FifthgetN.hat<-function(fit){
+ d<-predict(fit,type="state")$Predicted
+ a<-d*(1500*20/10000)
+ FifthN.hat<-c(FifthN.hat=sum(a))
+ return(FifthN.hat)
+ }
> Fifthpb<-parboot(hn_Fifth_Null,statistic=FifthgetN.hat,nsim=1000)
> Fifthpb
```
